# Supplementary figures and images for: Electrostatic lateral interactions drive ESCRT-III heteropolymer assembly (part 2 of 2)
Source: eLife. 2019 Jun 27;8:e46207. doi: 10.7554/eLife.46207 (PMC6663469; doi:10.7554/eLife.46207)

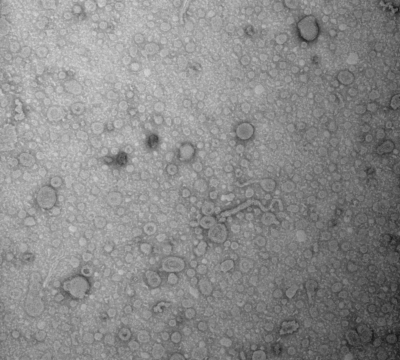

Supplement: Figure 5—figure supplement 2—source data 1. — The zip file contains images used to present the quantification in Figure 5—figure supplement 2C. For easy uploading and downloading, the sizes of the images have been downsized by 5.68 fold, using the Adjust size option in ImageJ, constraining the aspect ratio and using the bilinear interpolation option. In the images, 1 pixel equals 4.87 nm. [file elife-46207-fig5-figsupp2-data1.zip › Fig5 - Fig Supp 2C Source Data 1/Snf7R52E/60 min/11.tif]

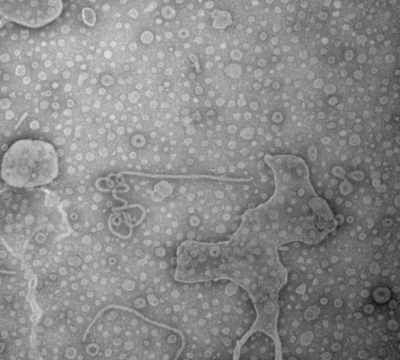

Supplement: Figure 5—figure supplement 2—source data 1. — The zip file contains images used to present the quantification in Figure 5—figure supplement 2C. For easy uploading and downloading, the sizes of the images have been downsized by 5.68 fold, using the Adjust size option in ImageJ, constraining the aspect ratio and using the bilinear interpolation option. In the images, 1 pixel equals 4.87 nm. [file elife-46207-fig5-figsupp2-data1.zip › Fig5 - Fig Supp 2C Source Data 1/Snf7R52E/60 min/12.tif]

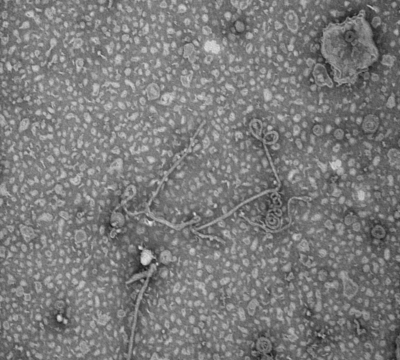

Supplement: Figure 5—figure supplement 2—source data 1. — The zip file contains images used to present the quantification in Figure 5—figure supplement 2C. For easy uploading and downloading, the sizes of the images have been downsized by 5.68 fold, using the Adjust size option in ImageJ, constraining the aspect ratio and using the bilinear interpolation option. In the images, 1 pixel equals 4.87 nm. [file elife-46207-fig5-figsupp2-data1.zip › Fig5 - Fig Supp 2C Source Data 1/Snf7R52E/60 min/13.tif]

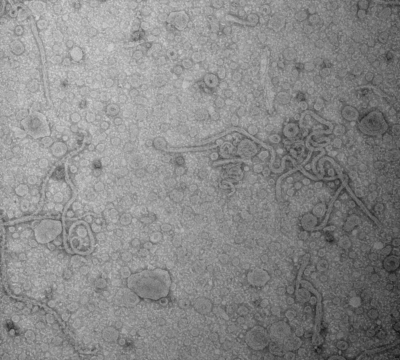

Supplement: Figure 5—figure supplement 2—source data 1. — The zip file contains images used to present the quantification in Figure 5—figure supplement 2C. For easy uploading and downloading, the sizes of the images have been downsized by 5.68 fold, using the Adjust size option in ImageJ, constraining the aspect ratio and using the bilinear interpolation option. In the images, 1 pixel equals 4.87 nm. [file elife-46207-fig5-figsupp2-data1.zip › Fig5 - Fig Supp 2C Source Data 1/Snf7R52E/60 min/14.tif]

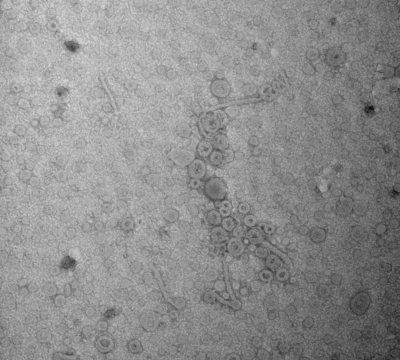

Supplement: Figure 5—figure supplement 2—source data 1. — The zip file contains images used to present the quantification in Figure 5—figure supplement 2C. For easy uploading and downloading, the sizes of the images have been downsized by 5.68 fold, using the Adjust size option in ImageJ, constraining the aspect ratio and using the bilinear interpolation option. In the images, 1 pixel equals 4.87 nm. [file elife-46207-fig5-figsupp2-data1.zip › Fig5 - Fig Supp 2C Source Data 1/Snf7R52E/60 min/15.tif]

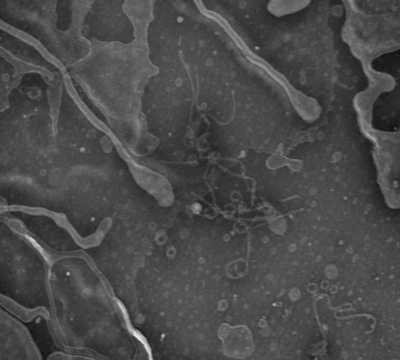

Supplement: Figure 5—figure supplement 2—source data 1. — The zip file contains images used to present the quantification in Figure 5—figure supplement 2C. For easy uploading and downloading, the sizes of the images have been downsized by 5.68 fold, using the Adjust size option in ImageJ, constraining the aspect ratio and using the bilinear interpolation option. In the images, 1 pixel equals 4.87 nm. [file elife-46207-fig5-figsupp2-data1.zip › Fig5 - Fig Supp 2C Source Data 1/Snf7R52E/60 min/16.tif]

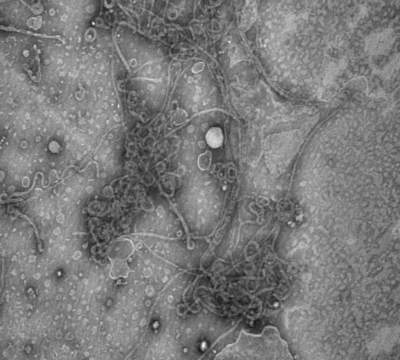

Supplement: Figure 5—figure supplement 2—source data 1. — The zip file contains images used to present the quantification in Figure 5—figure supplement 2C. For easy uploading and downloading, the sizes of the images have been downsized by 5.68 fold, using the Adjust size option in ImageJ, constraining the aspect ratio and using the bilinear interpolation option. In the images, 1 pixel equals 4.87 nm. [file elife-46207-fig5-figsupp2-data1.zip › Fig5 - Fig Supp 2C Source Data 1/Snf7R52E/60 min/18.tif]

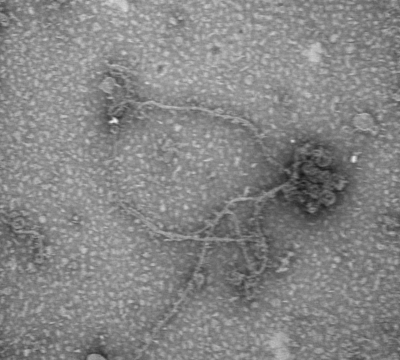

Supplement: Figure 5—figure supplement 2—source data 1. — The zip file contains images used to present the quantification in Figure 5—figure supplement 2C. For easy uploading and downloading, the sizes of the images have been downsized by 5.68 fold, using the Adjust size option in ImageJ, constraining the aspect ratio and using the bilinear interpolation option. In the images, 1 pixel equals 4.87 nm. [file elife-46207-fig5-figsupp2-data1.zip › Fig5 - Fig Supp 2C Source Data 1/Snf7R52E/60 min/19.tif]

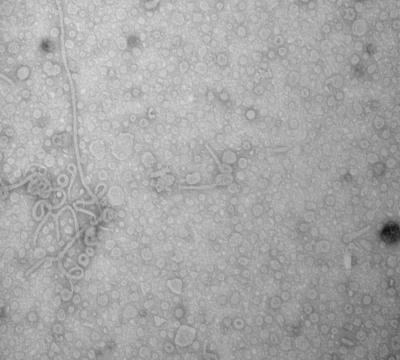

Supplement: Figure 5—figure supplement 2—source data 1. — The zip file contains images used to present the quantification in Figure 5—figure supplement 2C. For easy uploading and downloading, the sizes of the images have been downsized by 5.68 fold, using the Adjust size option in ImageJ, constraining the aspect ratio and using the bilinear interpolation option. In the images, 1 pixel equals 4.87 nm. [file elife-46207-fig5-figsupp2-data1.zip › Fig5 - Fig Supp 2C Source Data 1/Snf7R52E/60 min/2.tif]

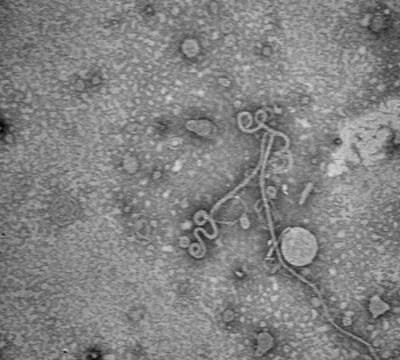

Supplement: Figure 5—figure supplement 2—source data 1. — The zip file contains images used to present the quantification in Figure 5—figure supplement 2C. For easy uploading and downloading, the sizes of the images have been downsized by 5.68 fold, using the Adjust size option in ImageJ, constraining the aspect ratio and using the bilinear interpolation option. In the images, 1 pixel equals 4.87 nm. [file elife-46207-fig5-figsupp2-data1.zip › Fig5 - Fig Supp 2C Source Data 1/Snf7R52E/60 min/20.tif]

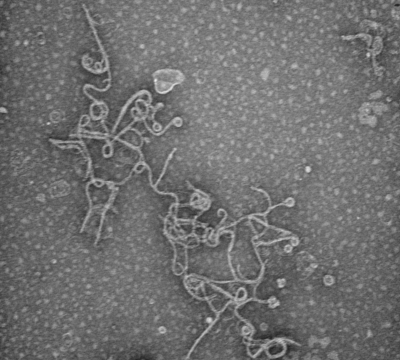

Supplement: Figure 5—figure supplement 2—source data 1. — The zip file contains images used to present the quantification in Figure 5—figure supplement 2C. For easy uploading and downloading, the sizes of the images have been downsized by 5.68 fold, using the Adjust size option in ImageJ, constraining the aspect ratio and using the bilinear interpolation option. In the images, 1 pixel equals 4.87 nm. [file elife-46207-fig5-figsupp2-data1.zip › Fig5 - Fig Supp 2C Source Data 1/Snf7R52E/60 min/21.tif]

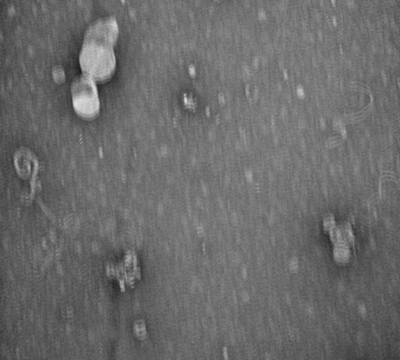

Supplement: Figure 5—figure supplement 2—source data 1. — The zip file contains images used to present the quantification in Figure 5—figure supplement 2C. For easy uploading and downloading, the sizes of the images have been downsized by 5.68 fold, using the Adjust size option in ImageJ, constraining the aspect ratio and using the bilinear interpolation option. In the images, 1 pixel equals 4.87 nm. [file elife-46207-fig5-figsupp2-data1.zip › Fig5 - Fig Supp 2C Source Data 1/Snf7R52E/60 min/22.tif]

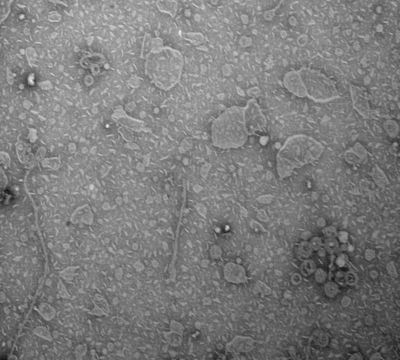

Supplement: Figure 5—figure supplement 2—source data 1. — The zip file contains images used to present the quantification in Figure 5—figure supplement 2C. For easy uploading and downloading, the sizes of the images have been downsized by 5.68 fold, using the Adjust size option in ImageJ, constraining the aspect ratio and using the bilinear interpolation option. In the images, 1 pixel equals 4.87 nm. [file elife-46207-fig5-figsupp2-data1.zip › Fig5 - Fig Supp 2C Source Data 1/Snf7R52E/60 min/23.tif]

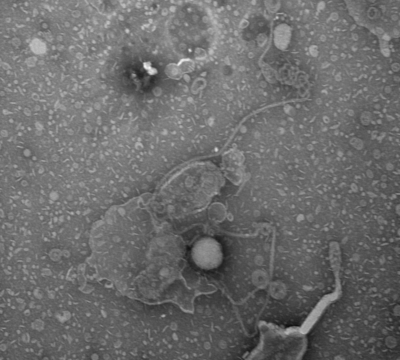

Supplement: Figure 5—figure supplement 2—source data 1. — The zip file contains images used to present the quantification in Figure 5—figure supplement 2C. For easy uploading and downloading, the sizes of the images have been downsized by 5.68 fold, using the Adjust size option in ImageJ, constraining the aspect ratio and using the bilinear interpolation option. In the images, 1 pixel equals 4.87 nm. [file elife-46207-fig5-figsupp2-data1.zip › Fig5 - Fig Supp 2C Source Data 1/Snf7R52E/60 min/24.tif]

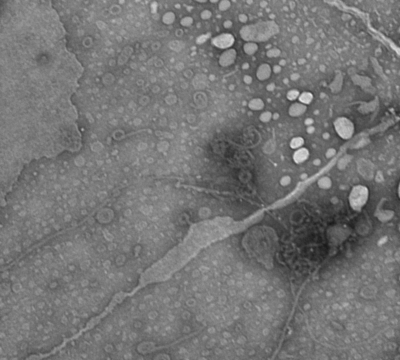

Supplement: Figure 5—figure supplement 2—source data 1. — The zip file contains images used to present the quantification in Figure 5—figure supplement 2C. For easy uploading and downloading, the sizes of the images have been downsized by 5.68 fold, using the Adjust size option in ImageJ, constraining the aspect ratio and using the bilinear interpolation option. In the images, 1 pixel equals 4.87 nm. [file elife-46207-fig5-figsupp2-data1.zip › Fig5 - Fig Supp 2C Source Data 1/Snf7R52E/60 min/25.tif]

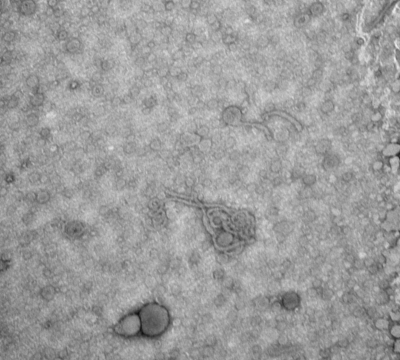

Supplement: Figure 5—figure supplement 2—source data 1. — The zip file contains images used to present the quantification in Figure 5—figure supplement 2C. For easy uploading and downloading, the sizes of the images have been downsized by 5.68 fold, using the Adjust size option in ImageJ, constraining the aspect ratio and using the bilinear interpolation option. In the images, 1 pixel equals 4.87 nm. [file elife-46207-fig5-figsupp2-data1.zip › Fig5 - Fig Supp 2C Source Data 1/Snf7R52E/60 min/26.tif]

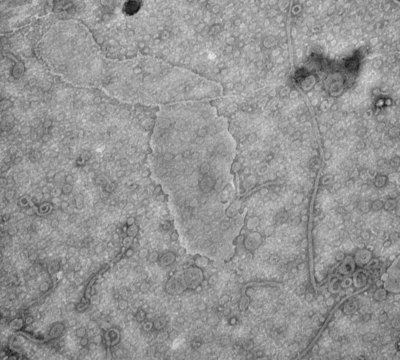

Supplement: Figure 5—figure supplement 2—source data 1. — The zip file contains images used to present the quantification in Figure 5—figure supplement 2C. For easy uploading and downloading, the sizes of the images have been downsized by 5.68 fold, using the Adjust size option in ImageJ, constraining the aspect ratio and using the bilinear interpolation option. In the images, 1 pixel equals 4.87 nm. [file elife-46207-fig5-figsupp2-data1.zip › Fig5 - Fig Supp 2C Source Data 1/Snf7R52E/60 min/27.tif]

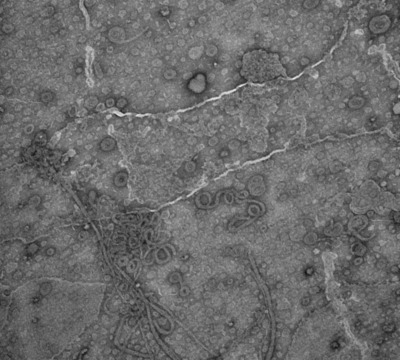

Supplement: Figure 5—figure supplement 2—source data 1. — The zip file contains images used to present the quantification in Figure 5—figure supplement 2C. For easy uploading and downloading, the sizes of the images have been downsized by 5.68 fold, using the Adjust size option in ImageJ, constraining the aspect ratio and using the bilinear interpolation option. In the images, 1 pixel equals 4.87 nm. [file elife-46207-fig5-figsupp2-data1.zip › Fig5 - Fig Supp 2C Source Data 1/Snf7R52E/60 min/28.tif]

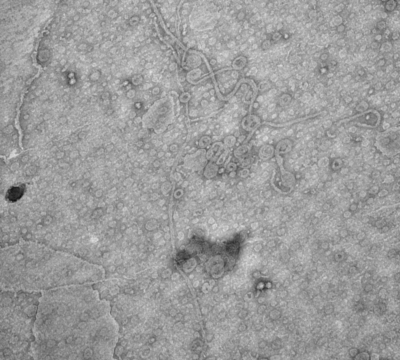

Supplement: Figure 5—figure supplement 2—source data 1. — The zip file contains images used to present the quantification in Figure 5—figure supplement 2C. For easy uploading and downloading, the sizes of the images have been downsized by 5.68 fold, using the Adjust size option in ImageJ, constraining the aspect ratio and using the bilinear interpolation option. In the images, 1 pixel equals 4.87 nm. [file elife-46207-fig5-figsupp2-data1.zip › Fig5 - Fig Supp 2C Source Data 1/Snf7R52E/60 min/29.tif]

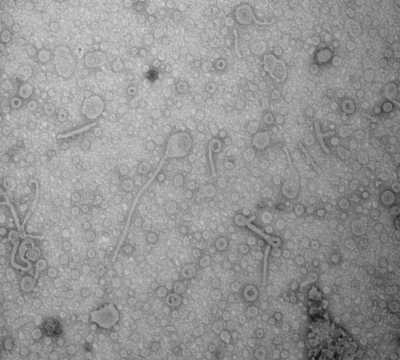

Supplement: Figure 5—figure supplement 2—source data 1. — The zip file contains images used to present the quantification in Figure 5—figure supplement 2C. For easy uploading and downloading, the sizes of the images have been downsized by 5.68 fold, using the Adjust size option in ImageJ, constraining the aspect ratio and using the bilinear interpolation option. In the images, 1 pixel equals 4.87 nm. [file elife-46207-fig5-figsupp2-data1.zip › Fig5 - Fig Supp 2C Source Data 1/Snf7R52E/60 min/3.tif]

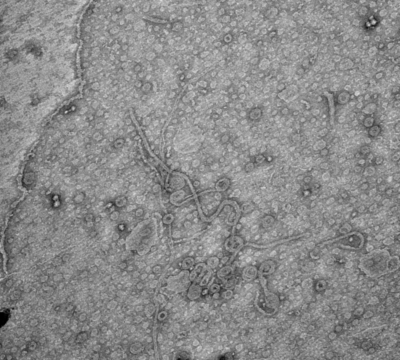

Supplement: Figure 5—figure supplement 2—source data 1. — The zip file contains images used to present the quantification in Figure 5—figure supplement 2C. For easy uploading and downloading, the sizes of the images have been downsized by 5.68 fold, using the Adjust size option in ImageJ, constraining the aspect ratio and using the bilinear interpolation option. In the images, 1 pixel equals 4.87 nm. [file elife-46207-fig5-figsupp2-data1.zip › Fig5 - Fig Supp 2C Source Data 1/Snf7R52E/60 min/30.tif]

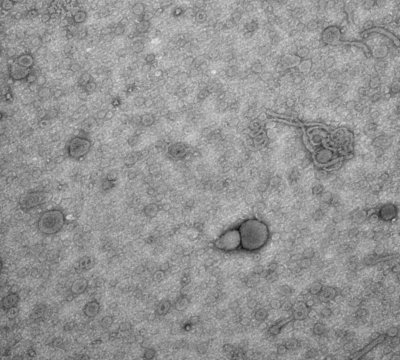

Supplement: Figure 5—figure supplement 2—source data 1. — The zip file contains images used to present the quantification in Figure 5—figure supplement 2C. For easy uploading and downloading, the sizes of the images have been downsized by 5.68 fold, using the Adjust size option in ImageJ, constraining the aspect ratio and using the bilinear interpolation option. In the images, 1 pixel equals 4.87 nm. [file elife-46207-fig5-figsupp2-data1.zip › Fig5 - Fig Supp 2C Source Data 1/Snf7R52E/60 min/31.tif]

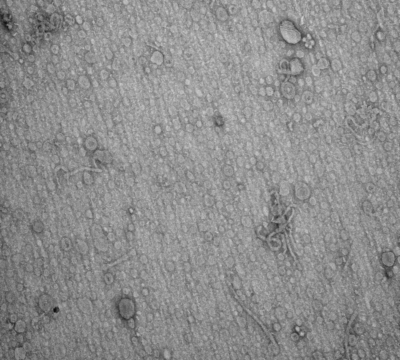

Supplement: Figure 5—figure supplement 2—source data 1. — The zip file contains images used to present the quantification in Figure 5—figure supplement 2C. For easy uploading and downloading, the sizes of the images have been downsized by 5.68 fold, using the Adjust size option in ImageJ, constraining the aspect ratio and using the bilinear interpolation option. In the images, 1 pixel equals 4.87 nm. [file elife-46207-fig5-figsupp2-data1.zip › Fig5 - Fig Supp 2C Source Data 1/Snf7R52E/60 min/32.tif]

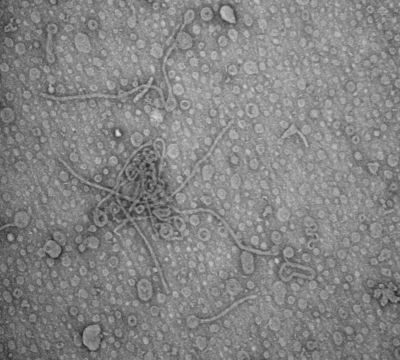

Supplement: Figure 5—figure supplement 2—source data 1. — The zip file contains images used to present the quantification in Figure 5—figure supplement 2C. For easy uploading and downloading, the sizes of the images have been downsized by 5.68 fold, using the Adjust size option in ImageJ, constraining the aspect ratio and using the bilinear interpolation option. In the images, 1 pixel equals 4.87 nm. [file elife-46207-fig5-figsupp2-data1.zip › Fig5 - Fig Supp 2C Source Data 1/Snf7R52E/60 min/33.tif]

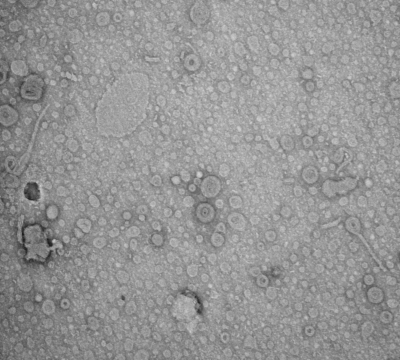

Supplement: Figure 5—figure supplement 2—source data 1. — The zip file contains images used to present the quantification in Figure 5—figure supplement 2C. For easy uploading and downloading, the sizes of the images have been downsized by 5.68 fold, using the Adjust size option in ImageJ, constraining the aspect ratio and using the bilinear interpolation option. In the images, 1 pixel equals 4.87 nm. [file elife-46207-fig5-figsupp2-data1.zip › Fig5 - Fig Supp 2C Source Data 1/Snf7R52E/60 min/34.tif]

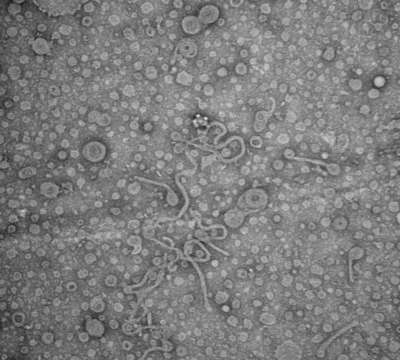

Supplement: Figure 5—figure supplement 2—source data 1. — The zip file contains images used to present the quantification in Figure 5—figure supplement 2C. For easy uploading and downloading, the sizes of the images have been downsized by 5.68 fold, using the Adjust size option in ImageJ, constraining the aspect ratio and using the bilinear interpolation option. In the images, 1 pixel equals 4.87 nm. [file elife-46207-fig5-figsupp2-data1.zip › Fig5 - Fig Supp 2C Source Data 1/Snf7R52E/60 min/35.tif]

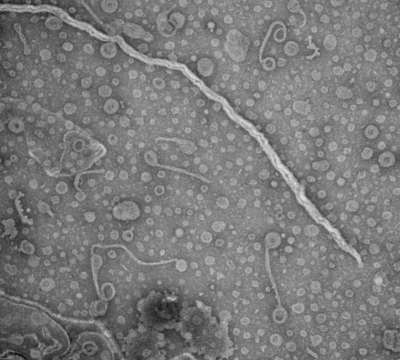

Supplement: Figure 5—figure supplement 2—source data 1. — The zip file contains images used to present the quantification in Figure 5—figure supplement 2C. For easy uploading and downloading, the sizes of the images have been downsized by 5.68 fold, using the Adjust size option in ImageJ, constraining the aspect ratio and using the bilinear interpolation option. In the images, 1 pixel equals 4.87 nm. [file elife-46207-fig5-figsupp2-data1.zip › Fig5 - Fig Supp 2C Source Data 1/Snf7R52E/60 min/36.tif]

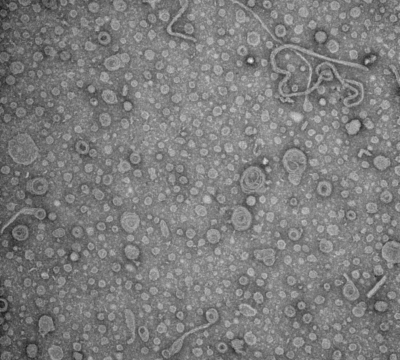

Supplement: Figure 5—figure supplement 2—source data 1. — The zip file contains images used to present the quantification in Figure 5—figure supplement 2C. For easy uploading and downloading, the sizes of the images have been downsized by 5.68 fold, using the Adjust size option in ImageJ, constraining the aspect ratio and using the bilinear interpolation option. In the images, 1 pixel equals 4.87 nm. [file elife-46207-fig5-figsupp2-data1.zip › Fig5 - Fig Supp 2C Source Data 1/Snf7R52E/60 min/37.tif]

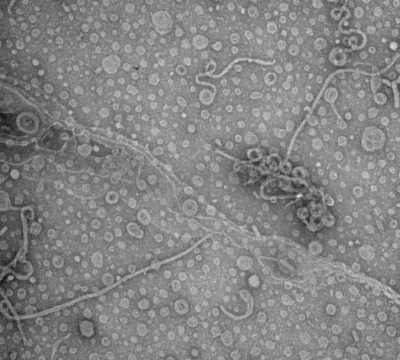

Supplement: Figure 5—figure supplement 2—source data 1. — The zip file contains images used to present the quantification in Figure 5—figure supplement 2C. For easy uploading and downloading, the sizes of the images have been downsized by 5.68 fold, using the Adjust size option in ImageJ, constraining the aspect ratio and using the bilinear interpolation option. In the images, 1 pixel equals 4.87 nm. [file elife-46207-fig5-figsupp2-data1.zip › Fig5 - Fig Supp 2C Source Data 1/Snf7R52E/60 min/38.tif]

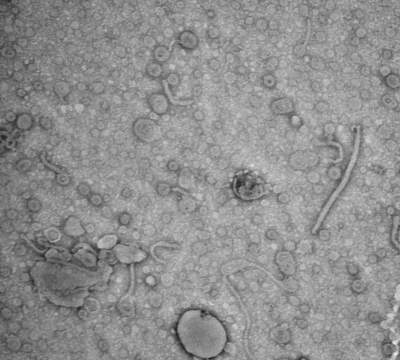

Supplement: Figure 5—figure supplement 2—source data 1. — The zip file contains images used to present the quantification in Figure 5—figure supplement 2C. For easy uploading and downloading, the sizes of the images have been downsized by 5.68 fold, using the Adjust size option in ImageJ, constraining the aspect ratio and using the bilinear interpolation option. In the images, 1 pixel equals 4.87 nm. [file elife-46207-fig5-figsupp2-data1.zip › Fig5 - Fig Supp 2C Source Data 1/Snf7R52E/60 min/39.tif]

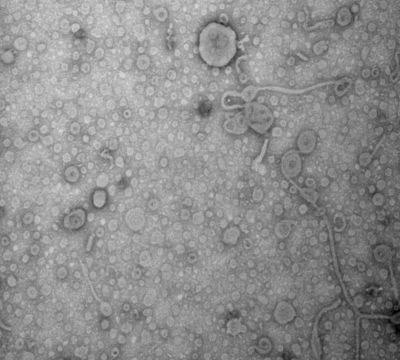

Supplement: Figure 5—figure supplement 2—source data 1. — The zip file contains images used to present the quantification in Figure 5—figure supplement 2C. For easy uploading and downloading, the sizes of the images have been downsized by 5.68 fold, using the Adjust size option in ImageJ, constraining the aspect ratio and using the bilinear interpolation option. In the images, 1 pixel equals 4.87 nm. [file elife-46207-fig5-figsupp2-data1.zip › Fig5 - Fig Supp 2C Source Data 1/Snf7R52E/60 min/4.tif]

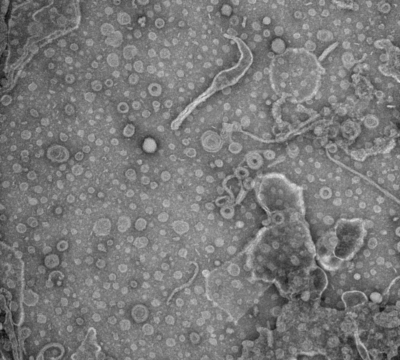

Supplement: Figure 5—figure supplement 2—source data 1. — The zip file contains images used to present the quantification in Figure 5—figure supplement 2C. For easy uploading and downloading, the sizes of the images have been downsized by 5.68 fold, using the Adjust size option in ImageJ, constraining the aspect ratio and using the bilinear interpolation option. In the images, 1 pixel equals 4.87 nm. [file elife-46207-fig5-figsupp2-data1.zip › Fig5 - Fig Supp 2C Source Data 1/Snf7R52E/60 min/40.tif]

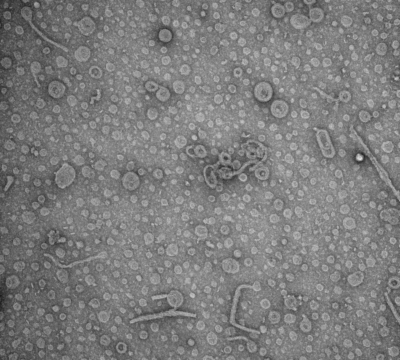

Supplement: Figure 5—figure supplement 2—source data 1. — The zip file contains images used to present the quantification in Figure 5—figure supplement 2C. For easy uploading and downloading, the sizes of the images have been downsized by 5.68 fold, using the Adjust size option in ImageJ, constraining the aspect ratio and using the bilinear interpolation option. In the images, 1 pixel equals 4.87 nm. [file elife-46207-fig5-figsupp2-data1.zip › Fig5 - Fig Supp 2C Source Data 1/Snf7R52E/60 min/41.tif]

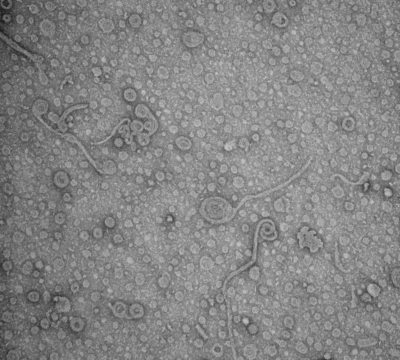

Supplement: Figure 5—figure supplement 2—source data 1. — The zip file contains images used to present the quantification in Figure 5—figure supplement 2C. For easy uploading and downloading, the sizes of the images have been downsized by 5.68 fold, using the Adjust size option in ImageJ, constraining the aspect ratio and using the bilinear interpolation option. In the images, 1 pixel equals 4.87 nm. [file elife-46207-fig5-figsupp2-data1.zip › Fig5 - Fig Supp 2C Source Data 1/Snf7R52E/60 min/42.tif]

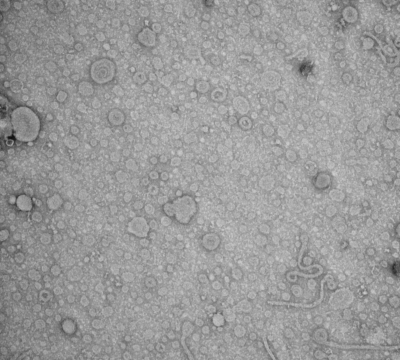

Supplement: Figure 5—figure supplement 2—source data 1. — The zip file contains images used to present the quantification in Figure 5—figure supplement 2C. For easy uploading and downloading, the sizes of the images have been downsized by 5.68 fold, using the Adjust size option in ImageJ, constraining the aspect ratio and using the bilinear interpolation option. In the images, 1 pixel equals 4.87 nm. [file elife-46207-fig5-figsupp2-data1.zip › Fig5 - Fig Supp 2C Source Data 1/Snf7R52E/60 min/43.tif]

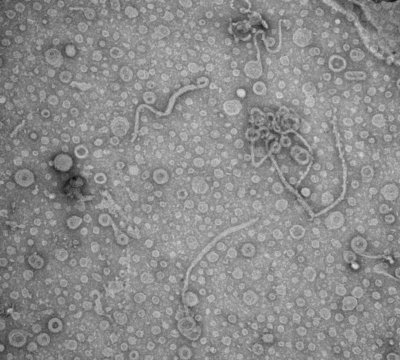

Supplement: Figure 5—figure supplement 2—source data 1. — The zip file contains images used to present the quantification in Figure 5—figure supplement 2C. For easy uploading and downloading, the sizes of the images have been downsized by 5.68 fold, using the Adjust size option in ImageJ, constraining the aspect ratio and using the bilinear interpolation option. In the images, 1 pixel equals 4.87 nm. [file elife-46207-fig5-figsupp2-data1.zip › Fig5 - Fig Supp 2C Source Data 1/Snf7R52E/60 min/44.tif]

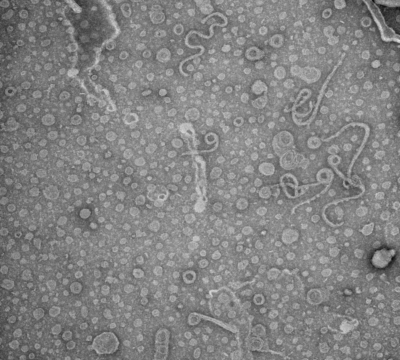

Supplement: Figure 5—figure supplement 2—source data 1. — The zip file contains images used to present the quantification in Figure 5—figure supplement 2C. For easy uploading and downloading, the sizes of the images have been downsized by 5.68 fold, using the Adjust size option in ImageJ, constraining the aspect ratio and using the bilinear interpolation option. In the images, 1 pixel equals 4.87 nm. [file elife-46207-fig5-figsupp2-data1.zip › Fig5 - Fig Supp 2C Source Data 1/Snf7R52E/60 min/45.tif]

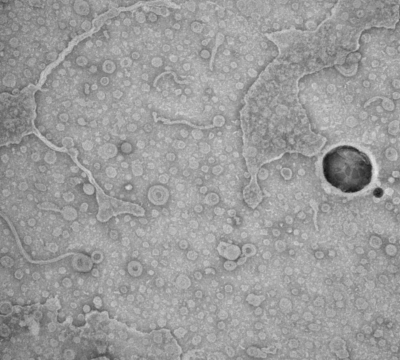

Supplement: Figure 5—figure supplement 2—source data 1. — The zip file contains images used to present the quantification in Figure 5—figure supplement 2C. For easy uploading and downloading, the sizes of the images have been downsized by 5.68 fold, using the Adjust size option in ImageJ, constraining the aspect ratio and using the bilinear interpolation option. In the images, 1 pixel equals 4.87 nm. [file elife-46207-fig5-figsupp2-data1.zip › Fig5 - Fig Supp 2C Source Data 1/Snf7R52E/60 min/46.tif]

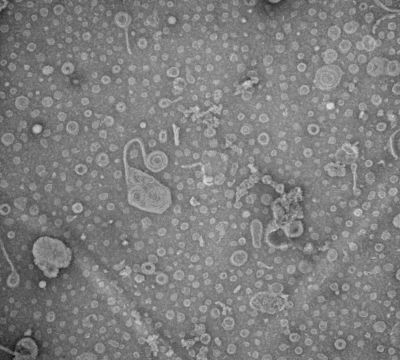

Supplement: Figure 5—figure supplement 2—source data 1. — The zip file contains images used to present the quantification in Figure 5—figure supplement 2C. For easy uploading and downloading, the sizes of the images have been downsized by 5.68 fold, using the Adjust size option in ImageJ, constraining the aspect ratio and using the bilinear interpolation option. In the images, 1 pixel equals 4.87 nm. [file elife-46207-fig5-figsupp2-data1.zip › Fig5 - Fig Supp 2C Source Data 1/Snf7R52E/60 min/47.tif]

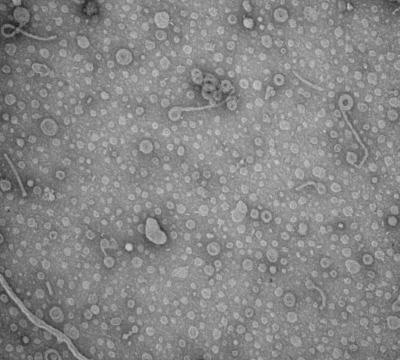

Supplement: Figure 5—figure supplement 2—source data 1. — The zip file contains images used to present the quantification in Figure 5—figure supplement 2C. For easy uploading and downloading, the sizes of the images have been downsized by 5.68 fold, using the Adjust size option in ImageJ, constraining the aspect ratio and using the bilinear interpolation option. In the images, 1 pixel equals 4.87 nm. [file elife-46207-fig5-figsupp2-data1.zip › Fig5 - Fig Supp 2C Source Data 1/Snf7R52E/60 min/48.tif]

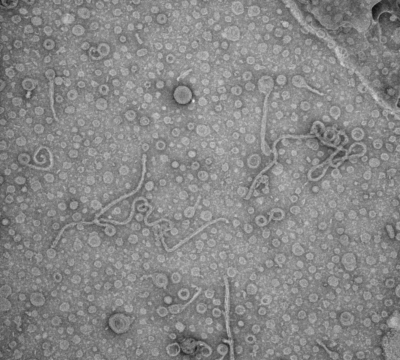

Supplement: Figure 5—figure supplement 2—source data 1. — The zip file contains images used to present the quantification in Figure 5—figure supplement 2C. For easy uploading and downloading, the sizes of the images have been downsized by 5.68 fold, using the Adjust size option in ImageJ, constraining the aspect ratio and using the bilinear interpolation option. In the images, 1 pixel equals 4.87 nm. [file elife-46207-fig5-figsupp2-data1.zip › Fig5 - Fig Supp 2C Source Data 1/Snf7R52E/60 min/49.tif]

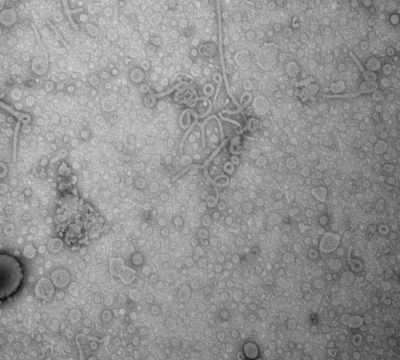

Supplement: Figure 5—figure supplement 2—source data 1. — The zip file contains images used to present the quantification in Figure 5—figure supplement 2C. For easy uploading and downloading, the sizes of the images have been downsized by 5.68 fold, using the Adjust size option in ImageJ, constraining the aspect ratio and using the bilinear interpolation option. In the images, 1 pixel equals 4.87 nm. [file elife-46207-fig5-figsupp2-data1.zip › Fig5 - Fig Supp 2C Source Data 1/Snf7R52E/60 min/5.tif]

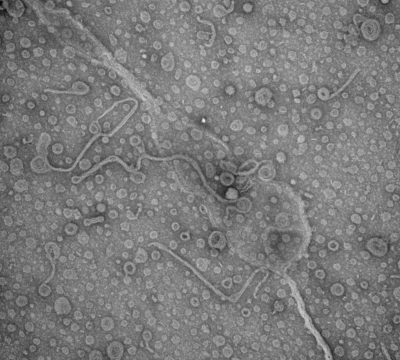

Supplement: Figure 5—figure supplement 2—source data 1. — The zip file contains images used to present the quantification in Figure 5—figure supplement 2C. For easy uploading and downloading, the sizes of the images have been downsized by 5.68 fold, using the Adjust size option in ImageJ, constraining the aspect ratio and using the bilinear interpolation option. In the images, 1 pixel equals 4.87 nm. [file elife-46207-fig5-figsupp2-data1.zip › Fig5 - Fig Supp 2C Source Data 1/Snf7R52E/60 min/50.tif]

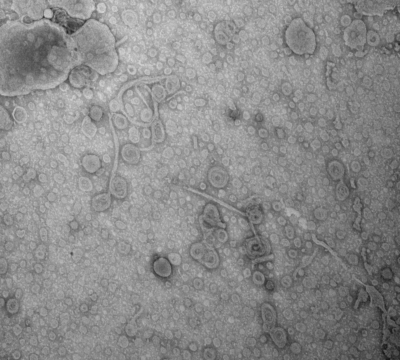

Supplement: Figure 5—figure supplement 2—source data 1. — The zip file contains images used to present the quantification in Figure 5—figure supplement 2C. For easy uploading and downloading, the sizes of the images have been downsized by 5.68 fold, using the Adjust size option in ImageJ, constraining the aspect ratio and using the bilinear interpolation option. In the images, 1 pixel equals 4.87 nm. [file elife-46207-fig5-figsupp2-data1.zip › Fig5 - Fig Supp 2C Source Data 1/Snf7R52E/60 min/6.tif]

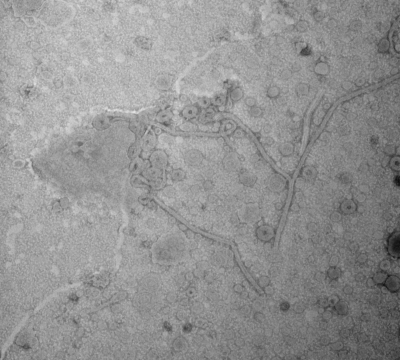

Supplement: Figure 5—figure supplement 2—source data 1. — The zip file contains images used to present the quantification in Figure 5—figure supplement 2C. For easy uploading and downloading, the sizes of the images have been downsized by 5.68 fold, using the Adjust size option in ImageJ, constraining the aspect ratio and using the bilinear interpolation option. In the images, 1 pixel equals 4.87 nm. [file elife-46207-fig5-figsupp2-data1.zip › Fig5 - Fig Supp 2C Source Data 1/Snf7R52E/60 min/7.tif]

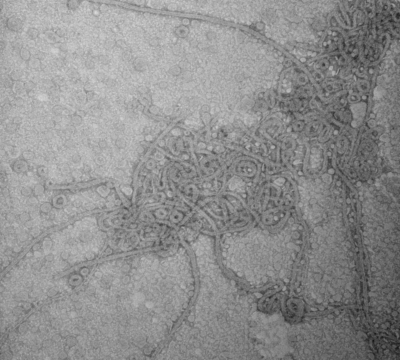

Supplement: Figure 5—figure supplement 2—source data 1. — The zip file contains images used to present the quantification in Figure 5—figure supplement 2C. For easy uploading and downloading, the sizes of the images have been downsized by 5.68 fold, using the Adjust size option in ImageJ, constraining the aspect ratio and using the bilinear interpolation option. In the images, 1 pixel equals 4.87 nm. [file elife-46207-fig5-figsupp2-data1.zip › Fig5 - Fig Supp 2C Source Data 1/Snf7R52E/60 min/8.tif]

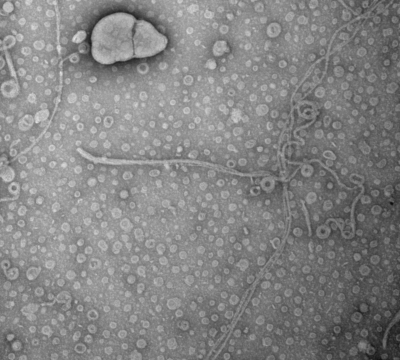

Supplement: Figure 5—figure supplement 2—source data 1. — The zip file contains images used to present the quantification in Figure 5—figure supplement 2C. For easy uploading and downloading, the sizes of the images have been downsized by 5.68 fold, using the Adjust size option in ImageJ, constraining the aspect ratio and using the bilinear interpolation option. In the images, 1 pixel equals 4.87 nm. [file elife-46207-fig5-figsupp2-data1.zip › Fig5 - Fig Supp 2C Source Data 1/Snf7R52E/60 min/9.tif]

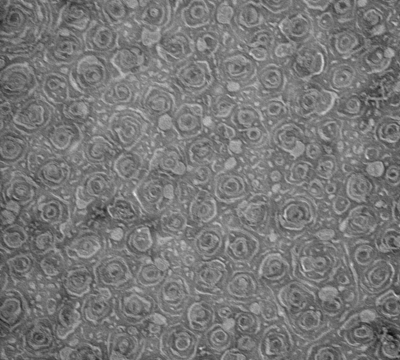

Supplement: Figure 5—figure supplement 2—source data 1. — The zip file contains images used to present the quantification in Figure 5—figure supplement 2C. For easy uploading and downloading, the sizes of the images have been downsized by 5.68 fold, using the Adjust size option in ImageJ, constraining the aspect ratio and using the bilinear interpolation option. In the images, 1 pixel equals 4.87 nm. [file elife-46207-fig5-figsupp2-data1.zip › Fig5 - Fig Supp 2C Source Data 1/Snf7R52E Vps24/10 min/1.tif]

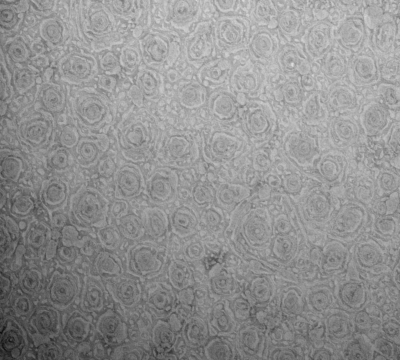

Supplement: Figure 5—figure supplement 2—source data 1. — The zip file contains images used to present the quantification in Figure 5—figure supplement 2C. For easy uploading and downloading, the sizes of the images have been downsized by 5.68 fold, using the Adjust size option in ImageJ, constraining the aspect ratio and using the bilinear interpolation option. In the images, 1 pixel equals 4.87 nm. [file elife-46207-fig5-figsupp2-data1.zip › Fig5 - Fig Supp 2C Source Data 1/Snf7R52E Vps24/10 min/10.tif]

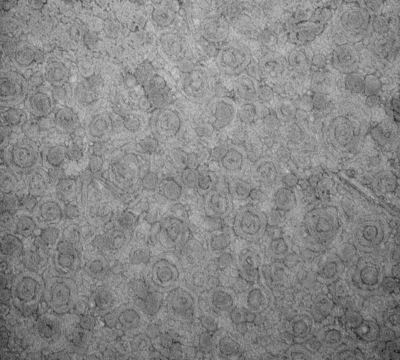

Supplement: Figure 5—figure supplement 2—source data 1. — The zip file contains images used to present the quantification in Figure 5—figure supplement 2C. For easy uploading and downloading, the sizes of the images have been downsized by 5.68 fold, using the Adjust size option in ImageJ, constraining the aspect ratio and using the bilinear interpolation option. In the images, 1 pixel equals 4.87 nm. [file elife-46207-fig5-figsupp2-data1.zip › Fig5 - Fig Supp 2C Source Data 1/Snf7R52E Vps24/10 min/11.tif]

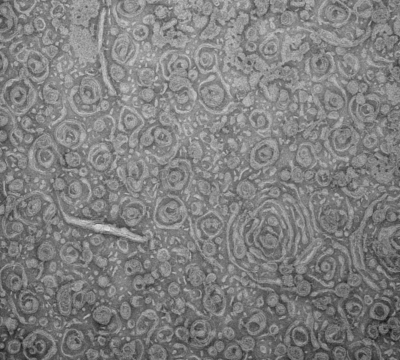

Supplement: Figure 5—figure supplement 2—source data 1. — The zip file contains images used to present the quantification in Figure 5—figure supplement 2C. For easy uploading and downloading, the sizes of the images have been downsized by 5.68 fold, using the Adjust size option in ImageJ, constraining the aspect ratio and using the bilinear interpolation option. In the images, 1 pixel equals 4.87 nm. [file elife-46207-fig5-figsupp2-data1.zip › Fig5 - Fig Supp 2C Source Data 1/Snf7R52E Vps24/10 min/12.tif]

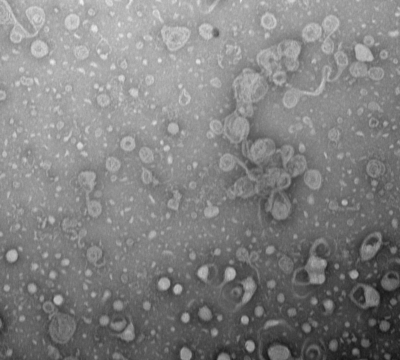

Supplement: Figure 5—figure supplement 2—source data 1. — The zip file contains images used to present the quantification in Figure 5—figure supplement 2C. For easy uploading and downloading, the sizes of the images have been downsized by 5.68 fold, using the Adjust size option in ImageJ, constraining the aspect ratio and using the bilinear interpolation option. In the images, 1 pixel equals 4.87 nm. [file elife-46207-fig5-figsupp2-data1.zip › Fig5 - Fig Supp 2C Source Data 1/Snf7R52E Vps24/10 min/13.tif]

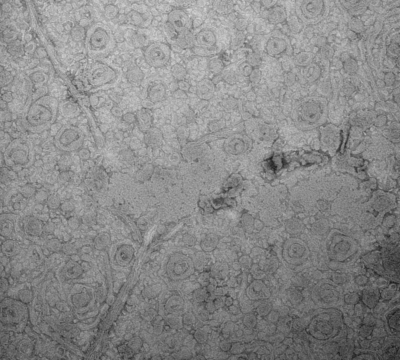

Supplement: Figure 5—figure supplement 2—source data 1. — The zip file contains images used to present the quantification in Figure 5—figure supplement 2C. For easy uploading and downloading, the sizes of the images have been downsized by 5.68 fold, using the Adjust size option in ImageJ, constraining the aspect ratio and using the bilinear interpolation option. In the images, 1 pixel equals 4.87 nm. [file elife-46207-fig5-figsupp2-data1.zip › Fig5 - Fig Supp 2C Source Data 1/Snf7R52E Vps24/10 min/14.tif]

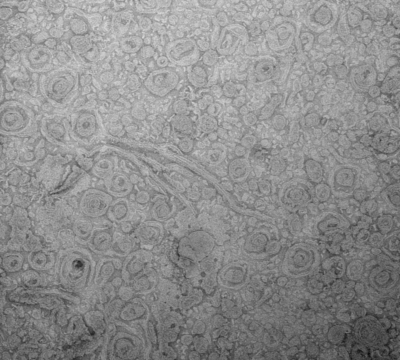

Supplement: Figure 5—figure supplement 2—source data 1. — The zip file contains images used to present the quantification in Figure 5—figure supplement 2C. For easy uploading and downloading, the sizes of the images have been downsized by 5.68 fold, using the Adjust size option in ImageJ, constraining the aspect ratio and using the bilinear interpolation option. In the images, 1 pixel equals 4.87 nm. [file elife-46207-fig5-figsupp2-data1.zip › Fig5 - Fig Supp 2C Source Data 1/Snf7R52E Vps24/10 min/15.tif]

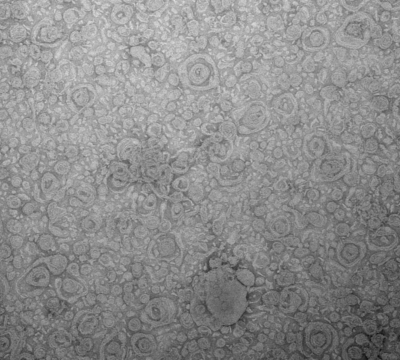

Supplement: Figure 5—figure supplement 2—source data 1. — The zip file contains images used to present the quantification in Figure 5—figure supplement 2C. For easy uploading and downloading, the sizes of the images have been downsized by 5.68 fold, using the Adjust size option in ImageJ, constraining the aspect ratio and using the bilinear interpolation option. In the images, 1 pixel equals 4.87 nm. [file elife-46207-fig5-figsupp2-data1.zip › Fig5 - Fig Supp 2C Source Data 1/Snf7R52E Vps24/10 min/16.tif]

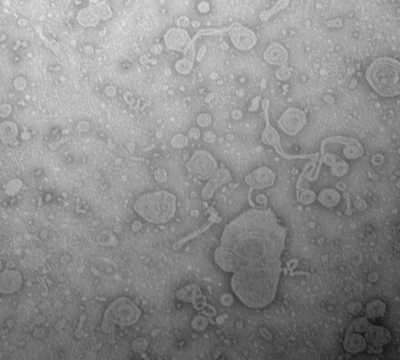

Supplement: Figure 5—figure supplement 2—source data 1. — The zip file contains images used to present the quantification in Figure 5—figure supplement 2C. For easy uploading and downloading, the sizes of the images have been downsized by 5.68 fold, using the Adjust size option in ImageJ, constraining the aspect ratio and using the bilinear interpolation option. In the images, 1 pixel equals 4.87 nm. [file elife-46207-fig5-figsupp2-data1.zip › Fig5 - Fig Supp 2C Source Data 1/Snf7R52E Vps24/10 min/18.tif]

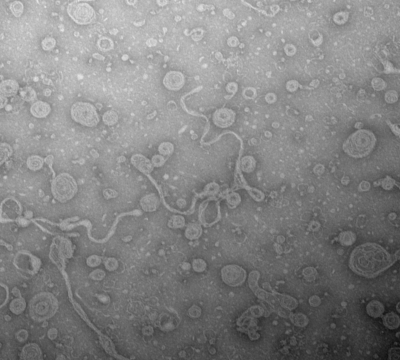

Supplement: Figure 5—figure supplement 2—source data 1. — The zip file contains images used to present the quantification in Figure 5—figure supplement 2C. For easy uploading and downloading, the sizes of the images have been downsized by 5.68 fold, using the Adjust size option in ImageJ, constraining the aspect ratio and using the bilinear interpolation option. In the images, 1 pixel equals 4.87 nm. [file elife-46207-fig5-figsupp2-data1.zip › Fig5 - Fig Supp 2C Source Data 1/Snf7R52E Vps24/10 min/19.tif]

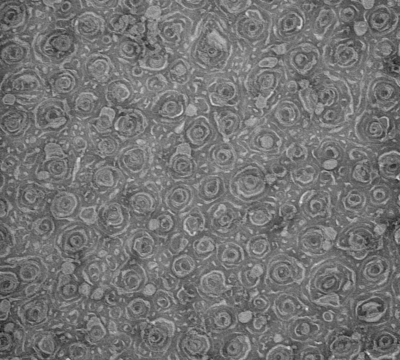

Supplement: Figure 5—figure supplement 2—source data 1. — The zip file contains images used to present the quantification in Figure 5—figure supplement 2C. For easy uploading and downloading, the sizes of the images have been downsized by 5.68 fold, using the Adjust size option in ImageJ, constraining the aspect ratio and using the bilinear interpolation option. In the images, 1 pixel equals 4.87 nm. [file elife-46207-fig5-figsupp2-data1.zip › Fig5 - Fig Supp 2C Source Data 1/Snf7R52E Vps24/10 min/2.tif]

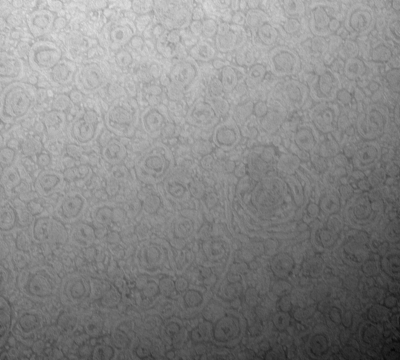

Supplement: Figure 5—figure supplement 2—source data 1. — The zip file contains images used to present the quantification in Figure 5—figure supplement 2C. For easy uploading and downloading, the sizes of the images have been downsized by 5.68 fold, using the Adjust size option in ImageJ, constraining the aspect ratio and using the bilinear interpolation option. In the images, 1 pixel equals 4.87 nm. [file elife-46207-fig5-figsupp2-data1.zip › Fig5 - Fig Supp 2C Source Data 1/Snf7R52E Vps24/10 min/20.tif]

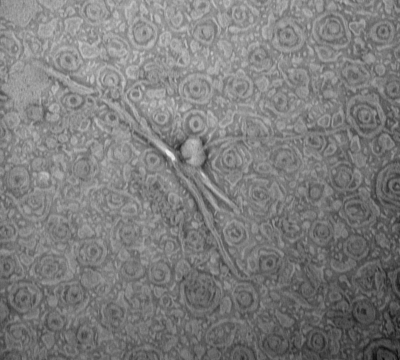

Supplement: Figure 5—figure supplement 2—source data 1. — The zip file contains images used to present the quantification in Figure 5—figure supplement 2C. For easy uploading and downloading, the sizes of the images have been downsized by 5.68 fold, using the Adjust size option in ImageJ, constraining the aspect ratio and using the bilinear interpolation option. In the images, 1 pixel equals 4.87 nm. [file elife-46207-fig5-figsupp2-data1.zip › Fig5 - Fig Supp 2C Source Data 1/Snf7R52E Vps24/10 min/21.tif]

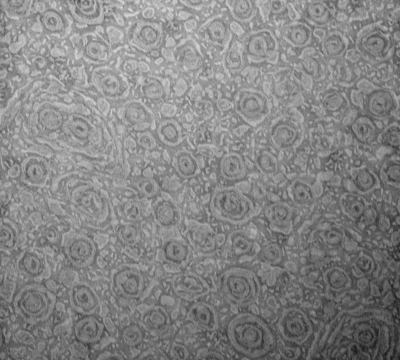

Supplement: Figure 5—figure supplement 2—source data 1. — The zip file contains images used to present the quantification in Figure 5—figure supplement 2C. For easy uploading and downloading, the sizes of the images have been downsized by 5.68 fold, using the Adjust size option in ImageJ, constraining the aspect ratio and using the bilinear interpolation option. In the images, 1 pixel equals 4.87 nm. [file elife-46207-fig5-figsupp2-data1.zip › Fig5 - Fig Supp 2C Source Data 1/Snf7R52E Vps24/10 min/22.tif]

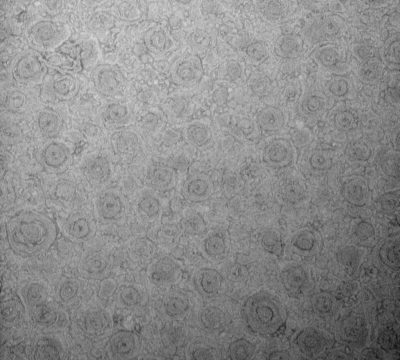

Supplement: Figure 5—figure supplement 2—source data 1. — The zip file contains images used to present the quantification in Figure 5—figure supplement 2C. For easy uploading and downloading, the sizes of the images have been downsized by 5.68 fold, using the Adjust size option in ImageJ, constraining the aspect ratio and using the bilinear interpolation option. In the images, 1 pixel equals 4.87 nm. [file elife-46207-fig5-figsupp2-data1.zip › Fig5 - Fig Supp 2C Source Data 1/Snf7R52E Vps24/10 min/23.tif]

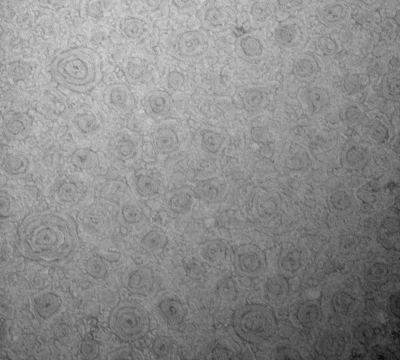

Supplement: Figure 5—figure supplement 2—source data 1. — The zip file contains images used to present the quantification in Figure 5—figure supplement 2C. For easy uploading and downloading, the sizes of the images have been downsized by 5.68 fold, using the Adjust size option in ImageJ, constraining the aspect ratio and using the bilinear interpolation option. In the images, 1 pixel equals 4.87 nm. [file elife-46207-fig5-figsupp2-data1.zip › Fig5 - Fig Supp 2C Source Data 1/Snf7R52E Vps24/10 min/24.tif]

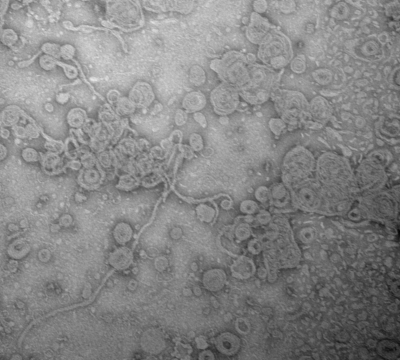

Supplement: Figure 5—figure supplement 2—source data 1. — The zip file contains images used to present the quantification in Figure 5—figure supplement 2C. For easy uploading and downloading, the sizes of the images have been downsized by 5.68 fold, using the Adjust size option in ImageJ, constraining the aspect ratio and using the bilinear interpolation option. In the images, 1 pixel equals 4.87 nm. [file elife-46207-fig5-figsupp2-data1.zip › Fig5 - Fig Supp 2C Source Data 1/Snf7R52E Vps24/10 min/25.tif]

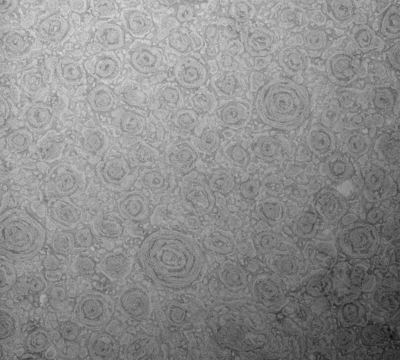

Supplement: Figure 5—figure supplement 2—source data 1. — The zip file contains images used to present the quantification in Figure 5—figure supplement 2C. For easy uploading and downloading, the sizes of the images have been downsized by 5.68 fold, using the Adjust size option in ImageJ, constraining the aspect ratio and using the bilinear interpolation option. In the images, 1 pixel equals 4.87 nm. [file elife-46207-fig5-figsupp2-data1.zip › Fig5 - Fig Supp 2C Source Data 1/Snf7R52E Vps24/10 min/26.tif]

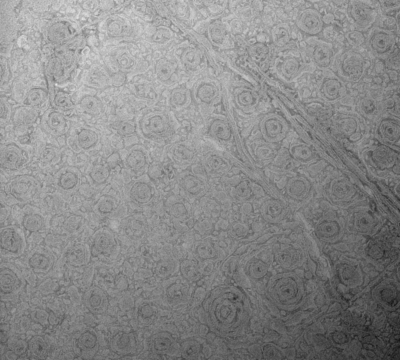

Supplement: Figure 5—figure supplement 2—source data 1. — The zip file contains images used to present the quantification in Figure 5—figure supplement 2C. For easy uploading and downloading, the sizes of the images have been downsized by 5.68 fold, using the Adjust size option in ImageJ, constraining the aspect ratio and using the bilinear interpolation option. In the images, 1 pixel equals 4.87 nm. [file elife-46207-fig5-figsupp2-data1.zip › Fig5 - Fig Supp 2C Source Data 1/Snf7R52E Vps24/10 min/27.tif]

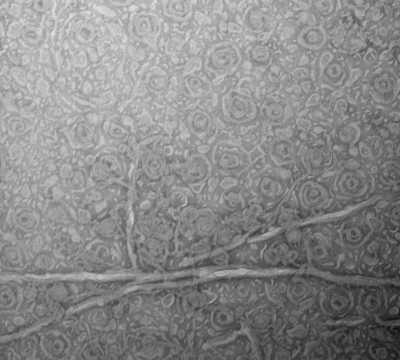

Supplement: Figure 5—figure supplement 2—source data 1. — The zip file contains images used to present the quantification in Figure 5—figure supplement 2C. For easy uploading and downloading, the sizes of the images have been downsized by 5.68 fold, using the Adjust size option in ImageJ, constraining the aspect ratio and using the bilinear interpolation option. In the images, 1 pixel equals 4.87 nm. [file elife-46207-fig5-figsupp2-data1.zip › Fig5 - Fig Supp 2C Source Data 1/Snf7R52E Vps24/10 min/28.tif]

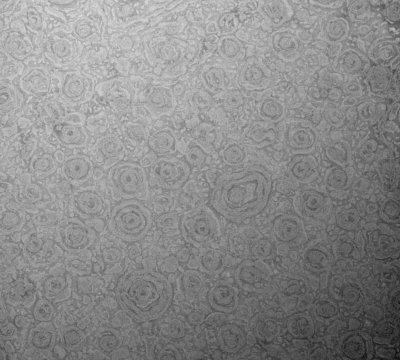

Supplement: Figure 5—figure supplement 2—source data 1. — The zip file contains images used to present the quantification in Figure 5—figure supplement 2C. For easy uploading and downloading, the sizes of the images have been downsized by 5.68 fold, using the Adjust size option in ImageJ, constraining the aspect ratio and using the bilinear interpolation option. In the images, 1 pixel equals 4.87 nm. [file elife-46207-fig5-figsupp2-data1.zip › Fig5 - Fig Supp 2C Source Data 1/Snf7R52E Vps24/10 min/29.tif]

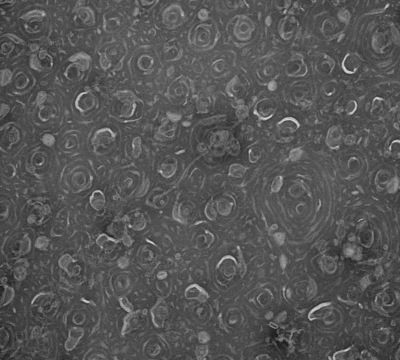

Supplement: Figure 5—figure supplement 2—source data 1. — The zip file contains images used to present the quantification in Figure 5—figure supplement 2C. For easy uploading and downloading, the sizes of the images have been downsized by 5.68 fold, using the Adjust size option in ImageJ, constraining the aspect ratio and using the bilinear interpolation option. In the images, 1 pixel equals 4.87 nm. [file elife-46207-fig5-figsupp2-data1.zip › Fig5 - Fig Supp 2C Source Data 1/Snf7R52E Vps24/10 min/3.tif]

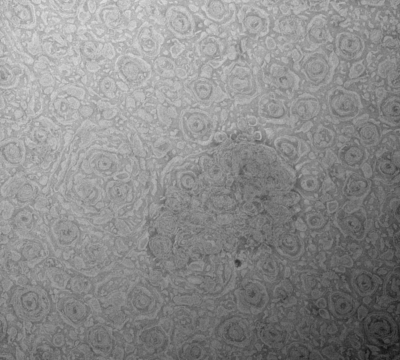

Supplement: Figure 5—figure supplement 2—source data 1. — The zip file contains images used to present the quantification in Figure 5—figure supplement 2C. For easy uploading and downloading, the sizes of the images have been downsized by 5.68 fold, using the Adjust size option in ImageJ, constraining the aspect ratio and using the bilinear interpolation option. In the images, 1 pixel equals 4.87 nm. [file elife-46207-fig5-figsupp2-data1.zip › Fig5 - Fig Supp 2C Source Data 1/Snf7R52E Vps24/10 min/30.tif]

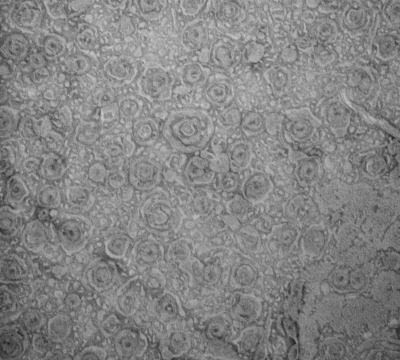

Supplement: Figure 5—figure supplement 2—source data 1. — The zip file contains images used to present the quantification in Figure 5—figure supplement 2C. For easy uploading and downloading, the sizes of the images have been downsized by 5.68 fold, using the Adjust size option in ImageJ, constraining the aspect ratio and using the bilinear interpolation option. In the images, 1 pixel equals 4.87 nm. [file elife-46207-fig5-figsupp2-data1.zip › Fig5 - Fig Supp 2C Source Data 1/Snf7R52E Vps24/10 min/31.tif]

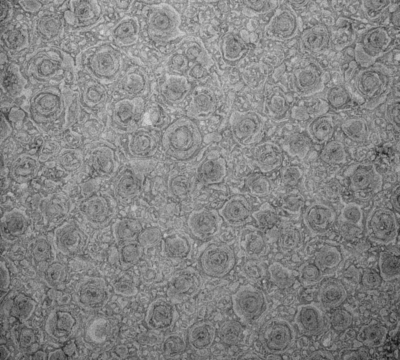

Supplement: Figure 5—figure supplement 2—source data 1. — The zip file contains images used to present the quantification in Figure 5—figure supplement 2C. For easy uploading and downloading, the sizes of the images have been downsized by 5.68 fold, using the Adjust size option in ImageJ, constraining the aspect ratio and using the bilinear interpolation option. In the images, 1 pixel equals 4.87 nm. [file elife-46207-fig5-figsupp2-data1.zip › Fig5 - Fig Supp 2C Source Data 1/Snf7R52E Vps24/10 min/32.tif]

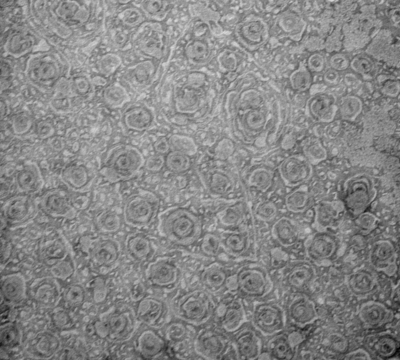

Supplement: Figure 5—figure supplement 2—source data 1. — The zip file contains images used to present the quantification in Figure 5—figure supplement 2C. For easy uploading and downloading, the sizes of the images have been downsized by 5.68 fold, using the Adjust size option in ImageJ, constraining the aspect ratio and using the bilinear interpolation option. In the images, 1 pixel equals 4.87 nm. [file elife-46207-fig5-figsupp2-data1.zip › Fig5 - Fig Supp 2C Source Data 1/Snf7R52E Vps24/10 min/33.tif]

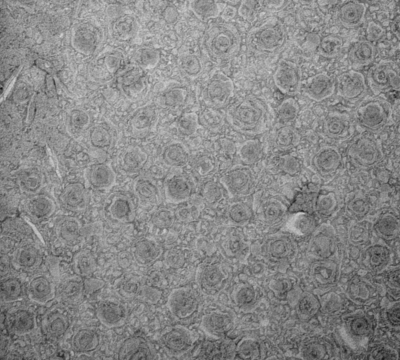

Supplement: Figure 5—figure supplement 2—source data 1. — The zip file contains images used to present the quantification in Figure 5—figure supplement 2C. For easy uploading and downloading, the sizes of the images have been downsized by 5.68 fold, using the Adjust size option in ImageJ, constraining the aspect ratio and using the bilinear interpolation option. In the images, 1 pixel equals 4.87 nm. [file elife-46207-fig5-figsupp2-data1.zip › Fig5 - Fig Supp 2C Source Data 1/Snf7R52E Vps24/10 min/34.tif]

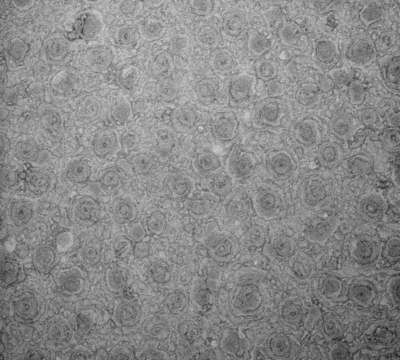

Supplement: Figure 5—figure supplement 2—source data 1. — The zip file contains images used to present the quantification in Figure 5—figure supplement 2C. For easy uploading and downloading, the sizes of the images have been downsized by 5.68 fold, using the Adjust size option in ImageJ, constraining the aspect ratio and using the bilinear interpolation option. In the images, 1 pixel equals 4.87 nm. [file elife-46207-fig5-figsupp2-data1.zip › Fig5 - Fig Supp 2C Source Data 1/Snf7R52E Vps24/10 min/35.tif]

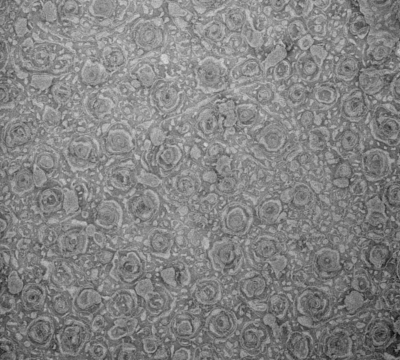

Supplement: Figure 5—figure supplement 2—source data 1. — The zip file contains images used to present the quantification in Figure 5—figure supplement 2C. For easy uploading and downloading, the sizes of the images have been downsized by 5.68 fold, using the Adjust size option in ImageJ, constraining the aspect ratio and using the bilinear interpolation option. In the images, 1 pixel equals 4.87 nm. [file elife-46207-fig5-figsupp2-data1.zip › Fig5 - Fig Supp 2C Source Data 1/Snf7R52E Vps24/10 min/36.tif]

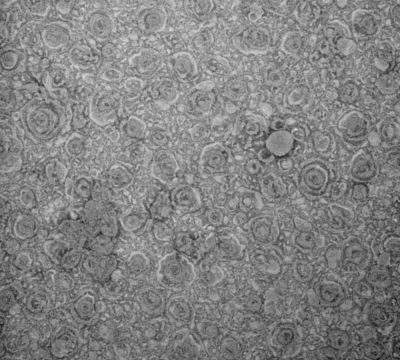

Supplement: Figure 5—figure supplement 2—source data 1. — The zip file contains images used to present the quantification in Figure 5—figure supplement 2C. For easy uploading and downloading, the sizes of the images have been downsized by 5.68 fold, using the Adjust size option in ImageJ, constraining the aspect ratio and using the bilinear interpolation option. In the images, 1 pixel equals 4.87 nm. [file elife-46207-fig5-figsupp2-data1.zip › Fig5 - Fig Supp 2C Source Data 1/Snf7R52E Vps24/10 min/37.tif]

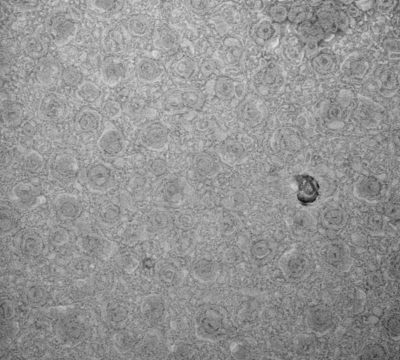

Supplement: Figure 5—figure supplement 2—source data 1. — The zip file contains images used to present the quantification in Figure 5—figure supplement 2C. For easy uploading and downloading, the sizes of the images have been downsized by 5.68 fold, using the Adjust size option in ImageJ, constraining the aspect ratio and using the bilinear interpolation option. In the images, 1 pixel equals 4.87 nm. [file elife-46207-fig5-figsupp2-data1.zip › Fig5 - Fig Supp 2C Source Data 1/Snf7R52E Vps24/10 min/38.tif]

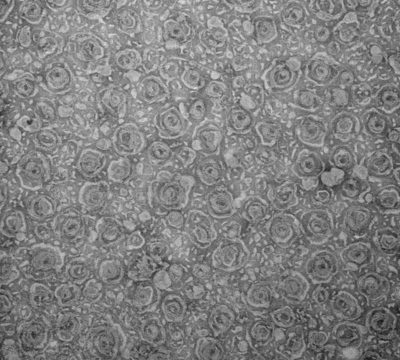

Supplement: Figure 5—figure supplement 2—source data 1. — The zip file contains images used to present the quantification in Figure 5—figure supplement 2C. For easy uploading and downloading, the sizes of the images have been downsized by 5.68 fold, using the Adjust size option in ImageJ, constraining the aspect ratio and using the bilinear interpolation option. In the images, 1 pixel equals 4.87 nm. [file elife-46207-fig5-figsupp2-data1.zip › Fig5 - Fig Supp 2C Source Data 1/Snf7R52E Vps24/10 min/39.tif]

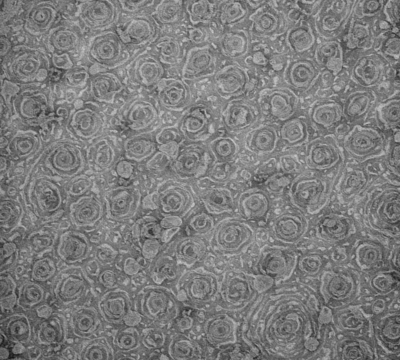

Supplement: Figure 5—figure supplement 2—source data 1. — The zip file contains images used to present the quantification in Figure 5—figure supplement 2C. For easy uploading and downloading, the sizes of the images have been downsized by 5.68 fold, using the Adjust size option in ImageJ, constraining the aspect ratio and using the bilinear interpolation option. In the images, 1 pixel equals 4.87 nm. [file elife-46207-fig5-figsupp2-data1.zip › Fig5 - Fig Supp 2C Source Data 1/Snf7R52E Vps24/10 min/4.tif]

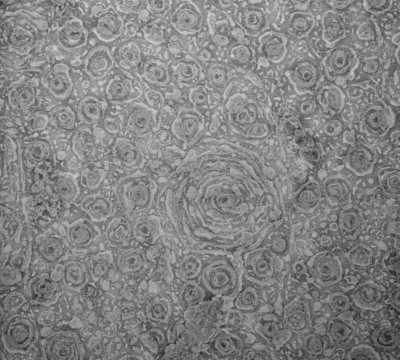

Supplement: Figure 5—figure supplement 2—source data 1. — The zip file contains images used to present the quantification in Figure 5—figure supplement 2C. For easy uploading and downloading, the sizes of the images have been downsized by 5.68 fold, using the Adjust size option in ImageJ, constraining the aspect ratio and using the bilinear interpolation option. In the images, 1 pixel equals 4.87 nm. [file elife-46207-fig5-figsupp2-data1.zip › Fig5 - Fig Supp 2C Source Data 1/Snf7R52E Vps24/10 min/40.tif]

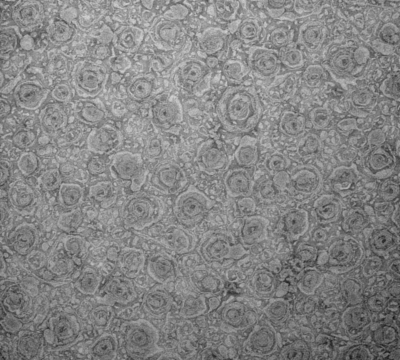

Supplement: Figure 5—figure supplement 2—source data 1. — The zip file contains images used to present the quantification in Figure 5—figure supplement 2C. For easy uploading and downloading, the sizes of the images have been downsized by 5.68 fold, using the Adjust size option in ImageJ, constraining the aspect ratio and using the bilinear interpolation option. In the images, 1 pixel equals 4.87 nm. [file elife-46207-fig5-figsupp2-data1.zip › Fig5 - Fig Supp 2C Source Data 1/Snf7R52E Vps24/10 min/41.tif]

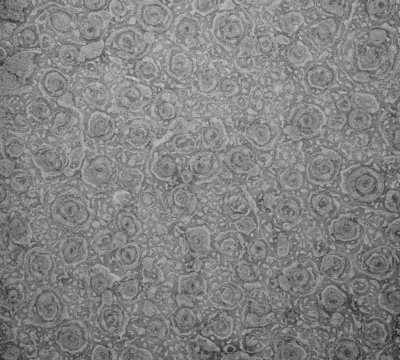

Supplement: Figure 5—figure supplement 2—source data 1. — The zip file contains images used to present the quantification in Figure 5—figure supplement 2C. For easy uploading and downloading, the sizes of the images have been downsized by 5.68 fold, using the Adjust size option in ImageJ, constraining the aspect ratio and using the bilinear interpolation option. In the images, 1 pixel equals 4.87 nm. [file elife-46207-fig5-figsupp2-data1.zip › Fig5 - Fig Supp 2C Source Data 1/Snf7R52E Vps24/10 min/42.tif]

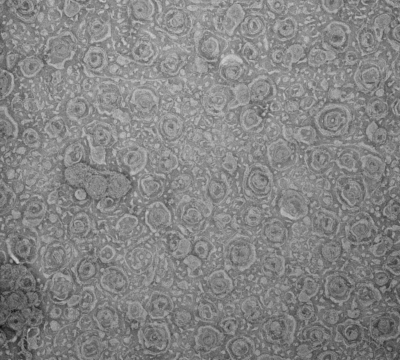

Supplement: Figure 5—figure supplement 2—source data 1. — The zip file contains images used to present the quantification in Figure 5—figure supplement 2C. For easy uploading and downloading, the sizes of the images have been downsized by 5.68 fold, using the Adjust size option in ImageJ, constraining the aspect ratio and using the bilinear interpolation option. In the images, 1 pixel equals 4.87 nm. [file elife-46207-fig5-figsupp2-data1.zip › Fig5 - Fig Supp 2C Source Data 1/Snf7R52E Vps24/10 min/43.tif]

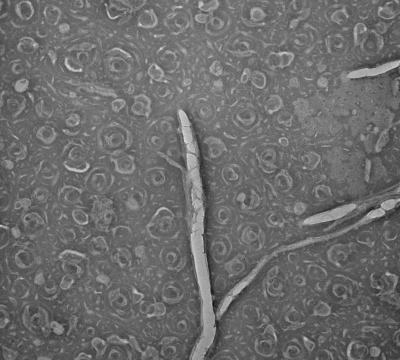

Supplement: Figure 5—figure supplement 2—source data 1. — The zip file contains images used to present the quantification in Figure 5—figure supplement 2C. For easy uploading and downloading, the sizes of the images have been downsized by 5.68 fold, using the Adjust size option in ImageJ, constraining the aspect ratio and using the bilinear interpolation option. In the images, 1 pixel equals 4.87 nm. [file elife-46207-fig5-figsupp2-data1.zip › Fig5 - Fig Supp 2C Source Data 1/Snf7R52E Vps24/10 min/44.tif]

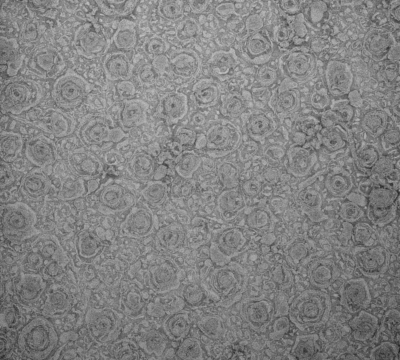

Supplement: Figure 5—figure supplement 2—source data 1. — The zip file contains images used to present the quantification in Figure 5—figure supplement 2C. For easy uploading and downloading, the sizes of the images have been downsized by 5.68 fold, using the Adjust size option in ImageJ, constraining the aspect ratio and using the bilinear interpolation option. In the images, 1 pixel equals 4.87 nm. [file elife-46207-fig5-figsupp2-data1.zip › Fig5 - Fig Supp 2C Source Data 1/Snf7R52E Vps24/10 min/45.tif]

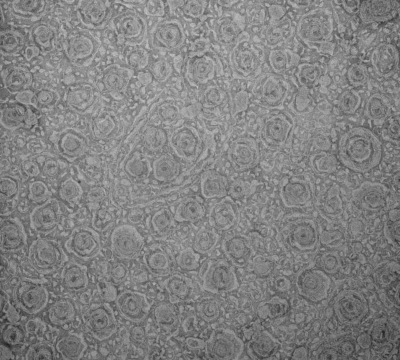

Supplement: Figure 5—figure supplement 2—source data 1. — The zip file contains images used to present the quantification in Figure 5—figure supplement 2C. For easy uploading and downloading, the sizes of the images have been downsized by 5.68 fold, using the Adjust size option in ImageJ, constraining the aspect ratio and using the bilinear interpolation option. In the images, 1 pixel equals 4.87 nm. [file elife-46207-fig5-figsupp2-data1.zip › Fig5 - Fig Supp 2C Source Data 1/Snf7R52E Vps24/10 min/46.tif]

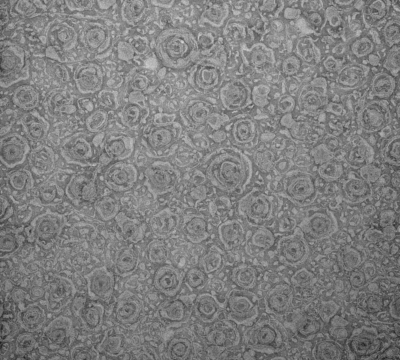

Supplement: Figure 5—figure supplement 2—source data 1. — The zip file contains images used to present the quantification in Figure 5—figure supplement 2C. For easy uploading and downloading, the sizes of the images have been downsized by 5.68 fold, using the Adjust size option in ImageJ, constraining the aspect ratio and using the bilinear interpolation option. In the images, 1 pixel equals 4.87 nm. [file elife-46207-fig5-figsupp2-data1.zip › Fig5 - Fig Supp 2C Source Data 1/Snf7R52E Vps24/10 min/47.tif]

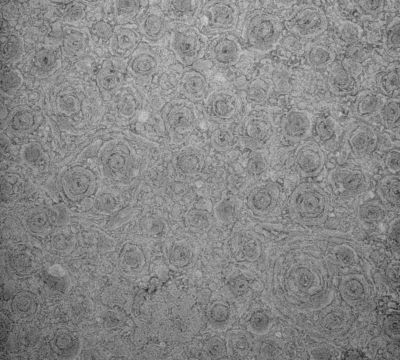

Supplement: Figure 5—figure supplement 2—source data 1. — The zip file contains images used to present the quantification in Figure 5—figure supplement 2C. For easy uploading and downloading, the sizes of the images have been downsized by 5.68 fold, using the Adjust size option in ImageJ, constraining the aspect ratio and using the bilinear interpolation option. In the images, 1 pixel equals 4.87 nm. [file elife-46207-fig5-figsupp2-data1.zip › Fig5 - Fig Supp 2C Source Data 1/Snf7R52E Vps24/10 min/48.tif]

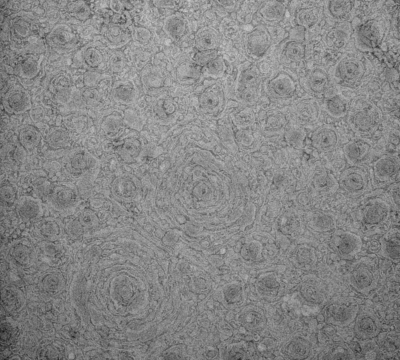

Supplement: Figure 5—figure supplement 2—source data 1. — The zip file contains images used to present the quantification in Figure 5—figure supplement 2C. For easy uploading and downloading, the sizes of the images have been downsized by 5.68 fold, using the Adjust size option in ImageJ, constraining the aspect ratio and using the bilinear interpolation option. In the images, 1 pixel equals 4.87 nm. [file elife-46207-fig5-figsupp2-data1.zip › Fig5 - Fig Supp 2C Source Data 1/Snf7R52E Vps24/10 min/49.tif]

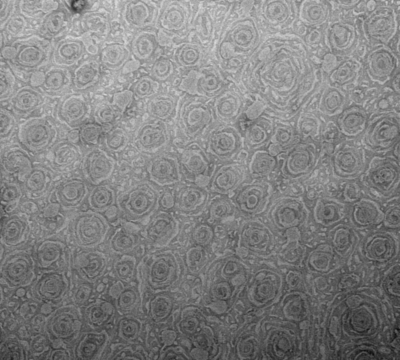

Supplement: Figure 5—figure supplement 2—source data 1. — The zip file contains images used to present the quantification in Figure 5—figure supplement 2C. For easy uploading and downloading, the sizes of the images have been downsized by 5.68 fold, using the Adjust size option in ImageJ, constraining the aspect ratio and using the bilinear interpolation option. In the images, 1 pixel equals 4.87 nm. [file elife-46207-fig5-figsupp2-data1.zip › Fig5 - Fig Supp 2C Source Data 1/Snf7R52E Vps24/10 min/5.tif]

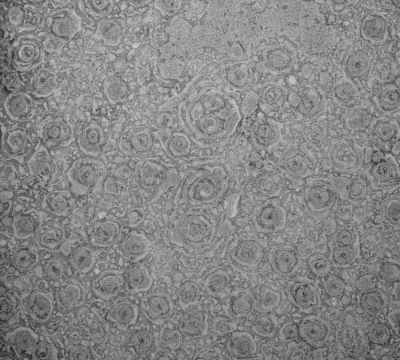

Supplement: Figure 5—figure supplement 2—source data 1. — The zip file contains images used to present the quantification in Figure 5—figure supplement 2C. For easy uploading and downloading, the sizes of the images have been downsized by 5.68 fold, using the Adjust size option in ImageJ, constraining the aspect ratio and using the bilinear interpolation option. In the images, 1 pixel equals 4.87 nm. [file elife-46207-fig5-figsupp2-data1.zip › Fig5 - Fig Supp 2C Source Data 1/Snf7R52E Vps24/10 min/50.tif]

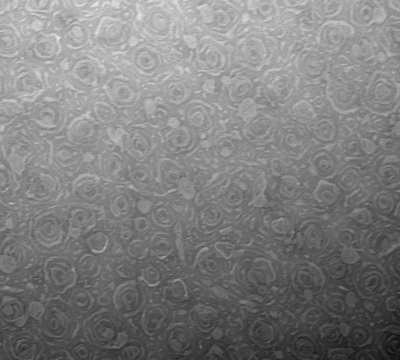

Supplement: Figure 5—figure supplement 2—source data 1. — The zip file contains images used to present the quantification in Figure 5—figure supplement 2C. For easy uploading and downloading, the sizes of the images have been downsized by 5.68 fold, using the Adjust size option in ImageJ, constraining the aspect ratio and using the bilinear interpolation option. In the images, 1 pixel equals 4.87 nm. [file elife-46207-fig5-figsupp2-data1.zip › Fig5 - Fig Supp 2C Source Data 1/Snf7R52E Vps24/10 min/6.tif]

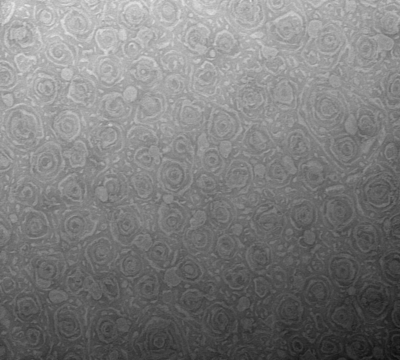

Supplement: Figure 5—figure supplement 2—source data 1. — The zip file contains images used to present the quantification in Figure 5—figure supplement 2C. For easy uploading and downloading, the sizes of the images have been downsized by 5.68 fold, using the Adjust size option in ImageJ, constraining the aspect ratio and using the bilinear interpolation option. In the images, 1 pixel equals 4.87 nm. [file elife-46207-fig5-figsupp2-data1.zip › Fig5 - Fig Supp 2C Source Data 1/Snf7R52E Vps24/10 min/7.tif]

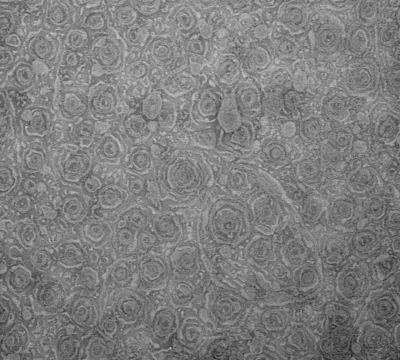

Supplement: Figure 5—figure supplement 2—source data 1. — The zip file contains images used to present the quantification in Figure 5—figure supplement 2C. For easy uploading and downloading, the sizes of the images have been downsized by 5.68 fold, using the Adjust size option in ImageJ, constraining the aspect ratio and using the bilinear interpolation option. In the images, 1 pixel equals 4.87 nm. [file elife-46207-fig5-figsupp2-data1.zip › Fig5 - Fig Supp 2C Source Data 1/Snf7R52E Vps24/10 min/8.tif]

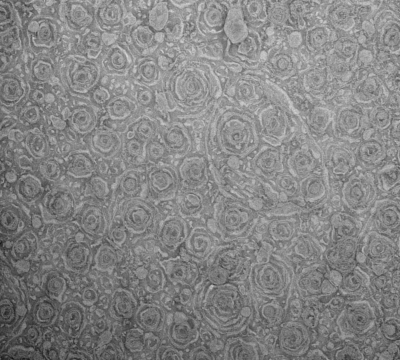

Supplement: Figure 5—figure supplement 2—source data 1. — The zip file contains images used to present the quantification in Figure 5—figure supplement 2C. For easy uploading and downloading, the sizes of the images have been downsized by 5.68 fold, using the Adjust size option in ImageJ, constraining the aspect ratio and using the bilinear interpolation option. In the images, 1 pixel equals 4.87 nm. [file elife-46207-fig5-figsupp2-data1.zip › Fig5 - Fig Supp 2C Source Data 1/Snf7R52E Vps24/10 min/9.tif]
