# Supplementary material for: Electrostatic lateral interactions drive ESCRT-III heteropolymer assembly
Source: eLife. 2019 Jun 27;8:e46207. doi: 10.7554/eLife.46207 (PMC6663469; doi:10.7554/eLife.46207)
Supplement: Supplementary file 1. [file elife-46207-supp1.docx]

| **Key Resources Table** |  |  |  |  |
| --- | --- | --- | --- | --- |
| **Reagent type (species) or resource** | **Designation** | **Source or reference** | **Identifiers** | **Additional information** |
| strain, (*Saccharomyces cerevisiae, Matα*) | WT | PMID:3062374 | SEY6210 | *(background strain) MATα ura3-52 his3-200 leu2-3,112 trp1-901 lys2-801 suc2-9* |
| strain, (*S. cerevisiae, Mata*) | WT | PMID:3062374 | SEY6210.1 | *(background strain) MATa leu2-3,112 ura3-52 his3-Δ200 trp1-Δ901 lys2-801 suc2-Δ9* |
| strain, (*S. cerevisiae, Mata*) | WT; *Mup-pHluorin* | PMID: 24139821 | NBY40 | *(SEY6210.1); MUP1-pHLUORIN::KANMX* |
| strain, (*S. cerevisiae, Mata*) | *snf7*Δ | PMID: 23063125 | NBY44 | *(SEY6210.1); snf7Δ::HIS3; MUP1-PHLUORIN::KAN* |
| strain, (*S. cerevisiae, Mata*) | *vps24*Δ | PMID: 24139821 | NBY47 | *(SEY6210.1); vps24Δ::HIS3; MUP1-pHLUORIN::KANMX* |
| strain, (*S. cerevisiae, Mata*) | *vps2*Δ | PMID: 24139821 | NBY69 | *(SEY6210.1); vps2Δ::HIS3 MUP1-pHLUORIN::KANMX* |
| strain, (*S. cerevisiae, Matα*) | *snf7*Δ *vps24*Δ | PMID: 26670543 | NBY56 | *(SEY6210); snf7Δ::HIS3 vps24Δ::HIS3 MUP1-pHLUORIN::KANMX* |
| strain, (*S. cerevisiae, Mata*) | *snf7*Δ *vps24-GFP* | This study | SBY57 | *(SEY6210.1); snf7Δ::Hph; vps24 LAP eGFP::His3 (made from vps24-LAP-eGFP reported in PMID 29019322)* |
| strain, (*S. cerevisiae, Mata*) | *snf7D131K* | This study | STY68 | *(SEY6210.1); snf7Δ::HIS3; snf7D131K::LEU2; MUP1-PHLUORIN::KAN (integration of snf7D131K into NBY44)* |
| strain, (*S. cerevisiae, Matα*) | *snf7D131K vps24*Δ | This study | STY70 | *(SEY6210); snf7Δ::HIS3; snf7D131K::LEU2; vps24Δ::HIS3; MUP1-PHLUORIN::KAN (integration of snf7D131K into NBY56)* |
| strain, (*S. cerevisiae, Matα*) | *snf7D131C* | This study | SBY83 | *(SEY6210), snf7::HIS3; vps24::HIS3; MUP1-pHLUORIN::KANMX; Snf7D131C::LEU2 (integration of snf7D131C into NBY56)* |
| strain, (*S. cerevisiae, Matα*) | *snf7D127KD131C* | This study | SBY172 | *(SEY6210), snf7::HIS3; vps24::HIS3; MUP1-pHLUORIN::KANMX; Snf7D127KD131C::URA3 (integration of snf7D127KD131C into NBY56)* |
| strain, (*S. cerevisiae, Matα*) | *snf7D127KE142KD131C* | This study | SBY173 | *(SEY6210), snf7::HIS3; vps24::HIS3; MUP1-pHLUORIN::KANMX; Snf7D127KE142KD131C::URA3 (integration of snf7D127KE142KD131C into NBY56)* |
| strain, (*S. cerevisiae, Mata*) | *snf7D131KR149D* | This study | SBY09 | *(SEY6210.1); snf7Δ::HIS3; snf7D131KR149D::LEU2; MUP1-PHLUORIN::KAN (integration of snf7D131KR149D into NBY44)* |
| strain, (*S. cerevisiae, Mata*) | *snf7 R25E H29E K36E* | This study | SBY28 | *(SEY6210.1); snf7 R25E H29E K36E::Leu2; snf7Δ::HIS3; MUP1-PHLUORIN::KAN (integration of snf7 R25E H29E K36E into NBY44)* |
| recombinant DNA reagent | Vector | PMID: 2659436 | pRS416 |  |
| recombinant DNA reagent | Vector | PMID: 2659436 | pRS414 |  |
| recombinant DNA reagent | Vector | PMID: 2659436 | pRS415 |  |
| recombinant DNA reagent | Snf7 (WT) | PMID: 23063125 | pRS416Snf7 | *S. cerevisiae* sequence |
| recombinant DNA reagent | Snf7 D127K | This study | pRS416Snf7D127K | Progenitor: pRS416 Snf7 (PMID: 23063125), Vector: pRS416 (PMID: 2659436), *S. cerevisiae* sequence |
| recombinant DNA reagent | Snf7 D131K | This study | pRS416Snf7D131K | Progenitor: pRS416 Snf7 (PMID: 23063125), Vector: pRS416 (PMID: 2659436), *S. cerevisiae* sequence |
| recombinant DNA reagent | Snf7 D127A | This study | pRS416Snf7D127A | Progenitor: pRS416 Snf7 (PMID: 23063125), Vector: pRS416 (PMID: 2659436), *S. cerevisiae* sequence |
| recombinant DNA reagent | Snf7 D131A | This study | pRS416Snf7D131A | Progenitor: pRS416 Snf7 (PMID: 23063125), Vector: pRS416 (PMID: 2659436), *S. cerevisiae* sequence |
| recombinant DNA reagent | Snf7 D127A D131A | This study | pRS416Snf7D127AD131A | Progenitor: pRS416 Snf7 (PMID: 23063125), Vector: pRS416 (PMID: 2659436), *S. cerevisiae* sequence |
| recombinant DNA reagent | Snf7 D122K | This study | pRS416Snf7D122K | Progenitor: pRS416 Snf7 (PMID: 23063125), Vector: pRS416 (PMID: 2659436), *S. cerevisiae* sequence |
| recombinant DNA reagent | Snf7 D124K | This study | pRS416Snf7D124K | Progenitor: pRS416 Snf7 (PMID: 23063125), Vector: pRS416 (PMID: 2659436), *S. cerevisiae* sequence |
| recombinant DNA reagent | Snf7 K125E | This study | pRS416Snf7K125E | Progenitor: pRS416 Snf7 (PMID: 23063125), Vector: pRS416 (PMID: 2659436), *S. cerevisiae* sequence |
| recombinant DNA reagent | Snf7 E128K | This study | pRS416Snf7E128K | Progenitor: pRS416 Snf7 (PMID: 23063125), Vector: pRS416 (PMID: 2659436), *S. cerevisiae* sequence |
| recombinant DNA reagent | Snf7 E132K | This study | pRS416Snf7E132K | Progenitor: pRS416 Snf7 (PMID: 23063125), Vector: pRS416 (PMID: 2659436), *S. cerevisiae* sequence |
| recombinant DNA reagent | Snf7 R134D | This study | pRS416Snf7R134D | Progenitor: pRS416 Snf7 (PMID: 23063125), Vector: pRS416 (PMID: 2659436), *S. cerevisiae* sequence |
| recombinant DNA reagent | Snf7 E135K | This study | pRS416Snf7E135K | Progenitor: pRS416 Snf7 (PMID: 23063125), Vector: pRS416 (PMID: 2659436), *S. cerevisiae* sequence |
| recombinant DNA reagent | Snf7 Q136E | This study | pRS416Snf7Q136E | Progenitor: pRS416 Snf7 (PMID: 23063125), Vector: pRS416 (PMID: 2659436), *S. cerevisiae* sequence |
| recombinant DNA reagent | Snf7 E138K | This study | pRS416Snf7E138K | Progenitor: pRS416 Snf7 (PMID: 23063125), Vector: pRS416 (PMID: 2659436), *S. cerevisiae* sequence |
| recombinant DNA reagent | Snf7 D141K | This study | pRS416Snf7D141K | Progenitor: pRS416 Snf7 (PMID: 23063125), Vector: pRS416 (PMID: 2659436), *S. cerevisiae* sequence |
| recombinant DNA reagent | Snf7 E142K | This study | pRS416Snf7E142K | Progenitor: pRS416 Snf7 (PMID: 23063125), Vector: pRS416 (PMID: 2659436), *S. cerevisiae* sequence |
| recombinant DNA reagent | Snf7 S144E | This study | pRS416Snf7S144E | Progenitor: pRS416 Snf7 (PMID: 23063125), Vector: pRS416 (PMID: 2659436), *S. cerevisiae* sequence |
| recombinant DNA reagent | Snf7 S148A | This study | pRS416Snf7S148A | Progenitor: pRS416 Snf7 (PMID: 23063125), Vector: pRS416 (PMID: 2659436), *S. cerevisiae* sequence |
| recombinant DNA reagent | Snf7 R149D | This study | pRS416Snf7R149D | Progenitor: pRS416 Snf7 (PMID: 23063125), Vector: pRS416 (PMID: 2659436), *S. cerevisiae* sequence |
| recombinant DNA reagent | Snf7 P150E | This study | pRS416Snf7P150E | Progenitor: pRS416 Snf7 (PMID: 23063125), Vector: pRS416 (PMID: 2659436), *S. cerevisiae* sequence |
| recombinant DNA reagent | Snf7 R52E | This study | pET28aH6SUMOSnf7R52E | Progenitor: pRS416 Snf7R52E (PMID: 2659436), Vector: pET28aH6SUMO (PMID: 26670543), *S. cerevisiae* sequence |
| recombinant DNA reagent | Snf7 R52E D131K | This study | pET28aH6SUMOSnf7R52ED131K | Progenitor: pET28aH6SUMOSnf7R52E, Vector: pET28aH6SUMO (PMID: 26670543), *S. cerevisiae* sequence |
| recombinant DNA reagent | Vps24 | This study | pET28aH6SUMOVps24 | Progenitor: pOPTVps24 (PMID: 18786397), Vector: pET28aH6SUMO (PMID: 26670543), *S. cerevisiae* protein sequence, DNA seq optimized for *E. coli* |
| recombinant DNA reagent | Vps2 | This study | pET28aH6SUMOVps2 | Progenitor: pOPTVps2 (PMID: 18786397), Vector: pET28aH6SUMO (PMID: 26670543) *S. cerevisiae* protein sequence, DNA seq optimized for *E. coli* |
| recombinant DNA reagent | Snf7 WT | This study | pET28aH6SUMOSnf7WT | Progenitor: pRS416 Snf7 (PMID: 23063125), Vector: pET28aH6SUMO (PMID: 26670543), *S. cerevisiae* sequence |
| recombinant DNA reagent | Snf7 K14E | This study | pRS416 Snf7K14E | Progenitor: pRS416 Snf7 (PMID: 23063125), Vector: pRS416 (PMID: 2659436), *S. cerevisiae* sequence |
| recombinant DNA reagent | Snf7 K16E | This study | pRS416 Snf7K16E | Progenitor: pRS416 Snf7 (PMID: 23063125), Vector: pRS416 (PMID: 2659436), *S. cerevisiae* sequence |
| recombinant DNA reagent | Snf7 K21E | This study | pRS416 Snf7K21E | Progenitor: pRS416 Snf7 (PMID: 23063125), Vector: pRS416 (PMID: 2659436), *S. cerevisiae* sequence |
| recombinant DNA reagent | Snf7 R25E | This study | pRS416 Snf7R25E | Progenitor: pRS416 Snf7 (PMID: 23063125), Vector: pRS416 (PMID: 2659436), *S. cerevisiae* sequence |
| recombinant DNA reagent | Snf7 K21ER25E | This study | pRS416 Snf7K21ER25E | Progenitor: pRS416 Snf7 (PMID: 23063125), Vector: pRS416 (PMID: 2659436), *S. cerevisiae* sequence |
| recombinant DNA reagent | Snf7 K21ED131K | This study | pRS416 Snf7K21ED131K | Progenitor: pRS416 Snf7 (PMID: 23063125), Vector: pRS416 (PMID: 2659436), *S. cerevisiae* sequence |
| recombinant DNA reagent | Snf7 R25ED131K | This study | pRS416 Snf7R25ED131K | Progenitor: pRS416 Snf7 (PMID: 23063125), Vector: pRS416 (PMID: 2659436), *S. cerevisiae* sequence |
| recombinant DNA reagent | Snf7 K21ER25ED131K | This study | pRS416 Snf7K21ER25ED131K | Progenitor: pRS416 Snf7 (PMID: 23063125), Vector: pRS416 (PMID: 2659436), *S. cerevisiae* sequence |
| recombinant DNA reagent | Snf7 D131K K14E | This study | pRS416 Snf7D131K K14E | Progenitor: pRS416 Snf7 (PMID: 23063125), Vector: pRS416 (PMID: 2659436), *S. cerevisiae* sequence |
| recombinant DNA reagent | Snf7 D131K K16E | This study | pRS416 Snf7D131K K16E | Progenitor: pRS416 Snf7 (PMID: 23063125), Vector: pRS416 (PMID: 2659436), *S. cerevisiae* sequence |
| recombinant DNA reagent | Snf7 (α0-α4) | PMID: 23063125 | pRS416 Snf7 core | *S. cerevisiae* sequence |
| recombinant DNA reagent | Snf7 (α0-α3) | PMID: 23063125 | pRS416 Snf7 core(α0-α3) | *S. cerevisiae* sequence |
| recombinant DNA reagent | Vps24WT | PMID: 23063125 | pRS 414 Vps24 | *S. cerevisiae* sequence |
| recombinant DNA reagent | Vps24Q16E | This study | pRS 414 Vps24 Q16E | Progenitor: pRS 414 Vps24 (PMID: 23063125), Vector: pRS414 (PMID: 2659436), *S. cerevisiae* sequence |
| recombinant DNA reagent | Vps24R19E | This study | pRS 414 Vps24 R19E | Progenitor: pRS 414 Vps24 (PMID: 23063125), Vector: pRS414 (PMID: 2659436), *S. cerevisiae* sequence |
| recombinant DNA reagent | Vps24-HA | This study | pRS414 Vps24WT-HA | Progenitor: pRS 414 Vps24 (PMID: 23063125), Vector: pRS414 (PMID: 2659436), *S. cerevisiae* sequence |
| recombinant DNA reagent | Vps24Q16C-HA | This study | pRS 414 Vps24 Q16C-HA | Progenitor: pRS 414 Vps24 (PMID: 23063125), Vector: pRS414 (PMID: 2659436), *S. cerevisiae* sequence |
| recombinant DNA reagent | Vps24R19C-HA | This study | pRS 414 Vps24 R19C-HA | Progenitor: pRS 414 Vps24 (PMID: 23063125), Vector: pRS414 (PMID: 2659436), *S. cerevisiae* sequence |
| recombinant DNA reagent | Vps24K26C-HA | This study | pRS 414 Vps24 K26C-HA | Progenitor: pRS 414 Vps24 (PMID: 23063125), Vector: pRS414 (PMID: 2659436), *S. cerevisiae* sequence |
| recombinant DNA reagent | Vps24Q16EQ17E | This study | pRS 414 Vps24 Q16EQ17E | Progenitor: pRS 414 Vps24 (PMID: 23063125), Vector: pRS414 (PMID: 2659436), *S. cerevisiae* sequence |
| recombinant DNA reagent | Vps24R18E | This study | pRS 414 Vps24 R18E | Progenitor: pRS 414 Vps24 (PMID: 23063125), Vector: pRS414 (PMID: 2659436), *S. cerevisiae* sequence |
| recombinant DNA reagent | Vps24R21E | This study | pRS 414 Vps24 R21E | Progenitor: pRS 414 Vps24 (PMID: 23063125), Vector: pRS414 (PMID: 2659436), *S. cerevisiae* sequence |
| recombinant DNA reagent | Vps24K26E | This study | pRS 414 Vps24 K26E | Progenitor: pRS 414 Vps24 (PMID: 23063125), Vector: pRS414 (PMID: 2659436), *S. cerevisiae* sequence |
| recombinant DNA reagent | Vps24K33E | This study | pRS 414 Vps24 K33E | Progenitor: pRS 414 Vps24 (PMID: 23063125), Vector: pRS414 (PMID: 2659436), *S. cerevisiae* sequence |
| recombinant DNA reagent | D131K R134D | This study | pRS 416 Snf7 D131K R134D | Progenitor: pRS416 Snf7 (PMID: 23063125), Vector: pRS416 (PMID: 2659436), *S. cerevisiae* sequence |
| recombinant DNA reagent | D131K R149D | This study | pRS 416 Snf7 D131K R149D | Progenitor: pRS416 Snf7 (PMID: 23063125), Vector: pRS416 (PMID: 2659436), *S. cerevisiae* sequence |
| recombinant DNA reagent | D127A D131A | This study | pRS 416 Snf7 D127A D131A | Progenitor: pRS416 Snf7 (PMID: 23063125), Vector: pRS416 (PMID: 2659436), *S. cerevisiae* sequence |
| recombinant DNA reagent | D127A D131A R134D | This study | pRS 416 Snf7 D127A D131A R134D | Progenitor: pRS416 Snf7 (PMID: 23063125), Vector: pRS416 (PMID: 2659436), *S. cerevisiae* sequence |
| recombinant DNA reagent | D127A D131A R149D | This study | pRS 416 Snf7 D127A D131A R149D | Progenitor: pRS416 Snf7 (PMID: 23063125), Vector: pRS416 (PMID: 2659436), *S. cerevisiae* sequence |
| recombinant DNA reagent | D131K R134A | This study | pRS 416 Snf7 D131K R134A | Progenitor: pRS416 Snf7 (PMID: 23063125), Vector: pRS416 (PMID: 2659436), *S. cerevisiae* sequence |
| recombinant DNA reagent | D131K R149A | This study | pRS 416 Snf7 D131K R149A | Progenitor: pRS416 Snf7 (PMID: 23063125), Vector: pRS416 (PMID: 2659436), *S. cerevisiae* sequence |
| recombinant DNA reagent | D127A D131A R134A | This study | pRS 416 Snf7 D127A D131A R134A | Progenitor: pRS416 Snf7 (PMID: 23063125), Vector: pRS416 (PMID: 2659436), *S. cerevisiae* sequence |
| recombinant DNA reagent | D127A D131A R149A | This study | pRS 416 Snf7 D127A D131A R149A | Progenitor: pRS416 Snf7 (PMID: 23063125), Vector: pRS416 (PMID: 2659436), *S. cerevisiae* sequence |
| recombinant DNA reagent | D131K K125E | This study | pRS 416 Snf7 D131K K125E | Progenitor: pRS416 Snf7 (PMID: 23063125), Vector: pRS416 (PMID: 2659436), *S. cerevisiae* sequence |
| recombinant DNA reagent | D131K Q136E | This study | pRS 416 Snf7 D131K Q136E | Progenitor: pRS416 Snf7 (PMID: 23063125), Vector: pRS416 (PMID: 2659436), *S. cerevisiae* sequence |
| recombinant DNA reagent | E142K R134D | This study | pRS 416 Snf7 E142K R134D | Progenitor: pRS416 Snf7 (PMID: 23063125), Vector: pRS416 (PMID: 2659436), *S. cerevisiae* sequence |
| recombinant DNA reagent | E142K R149D | This study | pRS 416 Snf7 E142K R149D | Progenitor: pRS416 Snf7 (PMID: 23063125), Vector: pRS416 (PMID: 2659436), *S. cerevisiae* sequence |
| recombinant DNA reagent | D127K R134D | This study | pRS 416 Snf7 D127K R134D | Progenitor: pRS416 Snf7 (PMID: 23063125), Vector: pRS416 (PMID: 2659436), *S. cerevisiae* sequence |
| recombinant DNA reagent | D127K R149D | This study | pRS 416 Snf7 D127K R149D | Progenitor: pRS416 Snf7 (PMID: 23063125), Vector: pRS416 (PMID: 2659436), *S. cerevisiae* sequence |
| recombinant DNA reagent | Vps20 | This study | pET28aH6SUMO Vps20 | Progenitor: pRS415 Vps20 (PMID: 27074665), Vector: pET28aH6SUMO (PMID: 26670543), S. cerevisiae sequence |
| recombinant DNA reagent | GST-Vps25 | PMID: 23063125 | pGEX6p1-Vps25 | *S. cerevisiae* sequence |
| recombinant DNA reagent | Vps24Q16E | This study | pET28aH6SUMO Vps24Q16E | Progenitor: pOPTVps24 (PMID: 18786397), Vector: pET28aH6SUMO (PMID: 26670543), *S. cerevisiae* protein sequence, DNA seq optimized for *E. coli* |
| recombinant DNA reagent | pCM189 | PMID: 9234672 | pCM189 |  |
| recombinant DNA reagent | pCM189 Vps24 (OE) | This study | pCM189 Vps24 | Progenitor: pRS 414 Vps24 (PMID: 23063125), Vector: pCM189 (PMID: 9234672), *S. cerevisiae* sequence |
| recombinant DNA reagent | pCM189 Vps2 (OE) | This study | pCM189 Vps2 | Progenitor: pRS 415 Vps2 (PMID: 23063125), Vector: pCM189 (PMID: 9234672), *S. cerevisiae* sequence |
| recombinant DNA reagent | Vps24 | This study | pRS 416 Vps24 | Progenitor: pRS 414 Vps24 (PMID: 23063125), Vector: pRS416 (PMID: 2659436), *S. cerevisiae* sequence |
| recombinant DNA reagent | Vps2 | This study | pRS 416 Vps2 | Progenitor: pRS 415 Vps2 (PMID: 23063125), Vector: pRS416 (PMID: 2659436), *S. cerevisiae* sequence |
| recombinant DNA reagent | Snf7 E95K E102K E109K | PMID: 26670543 | 414 Snf7 E95K E102K E109K | *S. cerevisiae* sequence |
| recombinant DNA reagent | Snf7 R25E H29E K36E | PMID: 26670543 | 414 Snf7 R25E H29E K36E | *S. cerevisiae* sequence |
| recombinant DNA reagent | Snf7 R25E K36E | PMID: 26670543 | 414 Snf7 R25E K36E | *S. cerevisiae* sequence |
| antibody | Rabbit polyclonal anti-GFP | [Torrey Pines Biolabs](https://scicrunch.org/resolver/RRID:AB_2313770) | (Torrey Pines Biolabs Cat# TP401, RRID:AB_2313770) | WB 1:2500 |
| antibody | Rabbit anti-G6PDH | [Sigma-Aldrich](https://scicrunch.org/resolver/RRID:AB_258454) | (Sigma-Aldrich Cat# A9521, RRID:AB_258454) | WB 1: 10000 |
| antibody | Mouse anti-PGK | Thermo Fisher | (Thermo Fisher Scientific Cat# 459250, RRID:AB_2532235) | WB 1:4000 |
| antibody | Mouse anti-HA | [Covance Research Products Inc](https://scicrunch.org/resolver/RRID:AB_2314672) | (Covance Research Products Inc Cat# MMS-101P, RRID:AB_2314672) | WB 1:2000 |
| antibody | Goat polyclonal -anti-Mouse 800/680 | LI-COR Biosciences | [LI-COR Biosciences Cat# 926–32210, RRID:AB_621842](https://scicrunch.org/resolver/AB_621842) | WB 1:10,000 |
| antibody | Goat polyclonal -anti-Rabbit 800/680 | LI-COR Biosciences | [LI-COR Biosciences Cat# 926–32211,RRID:AB_621843](https://scicrunch.org/resolver/AB_621843) | WB 1:10,000 |
| antibody | Rabbit polyclonal anti-Snf7 | PMID: 9606181 | anti-Snf7 Ab, S. cerevisiae Snf7 | WB 1:10000 |
| antibody | Rabbit polyclonal anti-Vps24 | PMID: 9606181 | anti-Vps24 Ab, S. cerevisiae Snf7 | WB 1:1000 |
| Software | Odyssey, Image Studio Lite | LI-COR Biosciences | (Image Studio Lite, RRID:SCR_013715) |  |
| Software | Mafft | <http://mafft.cbrc.jp/alignment/server/> | (MAFFT, RRID:SCR_011811) |  |
| Software | Jalview | [PMID:19151095](http://www.ncbi.nlm.nih.gov/pubmed/19151095) | (Jalview, RRID:SCR_006459) |  |
| Software | Modeller | https://salilab.org/modeller/download_installation.html | (MODELLER, RRID:SCR_008395) |  |
| Software | UCSF Chimera | [PMID:15264254](http://www.ncbi.nlm.nih.gov/pubmed/15264254) | (UCSF Chimera, RRID:SCR_004097) |  |
| Software | Fiji (ImageJ) | PMID: 22743772 | (Fiji, RRID:SCR_002285) |  |
| Other | BMOE | Thermo Fisher | Bismaleimidoethane, Cat. # 22323 | Made in DMSO, Final concentration used = 3.33 mM, Stock = 20 mM |
| Other | Canavanine | Sigma-Aldrich | L-Canavanine sulfate, Cat. # G8772 | Stock solution 5-10 mg/mL, made in water |
| Other | Cobalt resin | Clontech | TALON® Metal Affinity Resin, Cat #.635502 |  |
| Other | Glutathione resin | GE Healthcare | Glutathione Sepharose™ 4B, Cat. # 17-0756-01 |  |
| Other | HiTrap Q column | GE Healthcare | HiTrap Q Sepharose FF, Cat # 17-5053-01 |  |
| Other | SD200increase | GE Healthcare | Superdex 200 Increase 10/300 GL, Cat. # 28990944 |  |
| Other | EM grids | Electron Microscopy Sciences | Formvar Carbon Coated 200 Mesh Copper Grids, Cat. # FCF200-Cu |  |
| Other | Ammonium molybdate | Sigma-Aldrich | Ammonium molybdate 99.98%, Catalog#, 277908 | Made 2% (w/v) in water |
| Other | POPC | Avanti Polar Lipids | 16:0-18:1 PC (POPC), Cat.# 850457C |  |
| Other | POPS | Avanti Polar Lipids | 16:0-18:1 PC (POPC), Cat.# 840034 |  |
| Other | PI3P | Avanti Polar Lipids | 18:1 PI(3)P, Cat. # 850150 | Dissolved in 10% methanol, 90% chloroform, to make 0.25-1 mg/mL of solution |
